# Sheltered life beneath elytra: three new species of *Eutarsopolipus* (Acari, Heterostigmatina, Podapolipidae) parasitizing Australian ground beetles

**DOI:** 10.1051/parasite/2021069

**Published:** 2021-11-05

**Authors:** Alihan Katlav, Hamidreza Hajiqanbar, Markus Riegler, Owen D Seeman

**Affiliations:** 1 Hawkesbury Institute for the Environment, Western Sydney University Locked Bag 1797 Penrith NSW 2751 Australia; 2 Department of Entomology, Faculty of Agriculture, Tarbiat Modares University 14115-336 Tehran Iran; 3 Queensland Museum PO Box 3300 South Brisbane 4101 Australia

**Keywords:** Acari, Biodiversity, Trombidiformes, Carabidae, Symbiosis, Systematics

## Abstract

In this study, we conducted a summer sampling of carabid beetles in eastern Australia to identify their associated parasitic mites. Here, we describe three new species of the genus *Eutarsopolipus* from under the elytra (forewings) of three native carabid species (Coleoptera: Carabidae): *Eutarsopolipus paryavae* n. sp. (*pterostichi* group) from *Geoscaptus laevissimus* Chaudoir; *Eutarsopolipus pulcher* n. sp. (*leytei* group) from *Gnathaphanus pulcher* (Dejean); and *Eutarsopolipus chlaenii* n. sp. (*myzus* group) from *Chlaenius flaviguttatus* Macleay. We further provide an identification key of the world species of *pterostichi* and *leytei* species groups as well as closely related species of the *myzus* group possessing similar characters including short cheliceral stylets. The significant diversity of *Eutarsopolipus* recovered here suggests that the current knowledge about Australian podapolipid mites (specially *Eutarsopolipus*) is still in its infancy and deserves further study.

## Abstract (fr)

Dans cette étude, nous avons effectué un échantillonnage estival de carabes dans l’est de l’Australie pour identifier leurs acariens parasites associés. Nous décrivons trois nouvelles espèces du genre *Eutarsopolipus* sous les élytres (ailes antérieures) de trois espèces de carabes indigènes (Coleoptera : Carabidae) : *Eutarsopolipus paryavae* n. sp. (groupe *pterostichi*) de *Geoscaptus laevissimus* Chaudoir, *Eutarsopolipus pulcher* n. sp. (groupe *leytei*) de *Gnathaphanus pulcher* (Dejean) et *Eutarsopolipus chlaenii* n. sp. (groupe *myzus*) de *Chlaenius flaviguttatus* Macleay. Nous fournissons en outre une clé d’identification des espèces mondiales des groupes d’espèces *pterostichi* et *leytei* ainsi que des espèces étroitement apparentées du groupe *myzus* possédant des caractères similaires, y compris des stylets chélicéraux courts. La diversité importante des *Eutarsopolipus* collectés ici suggère que les connaissances actuelles sur les acariens podapolipidés australiens (en particulier *Eutarsopolipus*) en sont encore à leurs balbutiements et méritent une étude plus approfondie.

## Introduction

Beetles are among the most successful animals on the planet, accounting for about 25% of described species [10, 46]. Their success is partly attributed to their modified, sclerotized forewings, known as elytra, that protect their body against physical damage, desiccation, predation and thermal stress, enabling them to occupy a wide range of ecological niches [33, 48]. The subelytral space serves as a suitable microhabitat for a broad range of organisms such as mites, pseudoscorpions and nematodes that occupy this niche temporarily or permanently [6, 36, 37]. Some mites have evolved to be permanent ectoparasites in the subelytral spaces of beetles, imbibing beetle haemolymph using piercing stylets [2, 7]. This parasitic association sometimes occurs in one part of a mite’s life cycle. For example, in Parasitengona (Acariformes: Prostigmata), larvae are parasites of many insects and are sometimes found under the elytra of terrestrial and aquatic beetles, while the nymphs and adults are free-living predators of immature stages of small arthropods [51, 52]. However, some taxa represent evolutionary transitions from phoresy towards parasitism, as in a few canestrinioid mites (Astigmata) in which deutonymphs remain phoretic on the thoracic venter of some carabid beetles, whereas the other stages (feeding stages) are subelytral parasites of the same hosts [15, 49]. Some other groups are real parasites with their abbreviated life stages all occurring on the host’s body surface [47]. Such subelytral parasitic associations with beetles have evolved independently in many Astigmata (e.g. most members of Canestrinioidea), Mesostigmata (e.g. several members of Gamasina) and Prostigmata (e.g. several members of Raphignathina and Heterostigmatina) [14, 16, 35, 38].

The cohort Heterostigmatina (Acariformes: Prostigmata) is a large group of morphologically diverse mites, among which numerous species are associated with arthropods [49]. Some species are subelytral symbionts of various beetles with their associations varying from facultative or obligate phoresy to parasitoidism or parasitism [25, 28, 30, 31]. Several species are potential biocontrol agents against pest beetles. For example, the species of the families Pyemotidae and Acarophenacidae are known as insect ectoparasitoids, with the former mostly attacking juvenile stages of bark beetles and stored-product beetles and the latter egg ectoparasitoids of various beetle families [8, 25, 29, 50].

All members of the family Podapolipidae are specialized obligate external (and rarely internal) parasites of various insects [18], among which at least 20 genera are subelytral ectoparasites of different beetle families, mainly Carabidae, Chrysomelidae, Coccinellidae, and Scarabaeidae [21, 23, 38, 45]. These mites are sexually transmitted, i.e. the motile stages of the mite (larvae or adult females) move from one host individual to another during copulation [17]. Parasitisation with these mites can negatively affect host fitness. For example, in some ladybirds, individuals parasitised with *Coccipolipus* suffer lower fecundity and egg viability [17] and sometimes reduced longevity [40]. Beyond this, these mites can modify host sexual and behavioural traits to boost their transmission success among individual hosts [1]. For example, in the milk weed leaf beetle, males parasitized by *Chrysomelobia* tend to more frequently contact other males, and are more successful in mating competition compared to unparasitised males; and this facilitates the mite’s higher transmission rate [1].

Four genera of Podapolipidae are exclusively associated with carabid beetles: *Dorsipes* (22 species), *Eutarsopolipus* (99 species), *Ovacarus* (3 species) and *Regenpolipus* (5 species) [11, 13, 19, 26, 27, 44]. Apart from *Ovacarus*, which is an endoparasite of the reproductive tracts of some carabids, the rest are subelytral ectoparasites [11]. Species of *Eutarsopolipus* are versatile in morphology and are currently grouped into ten species groups [42]. Most of the species are specific to a single host species. However, a few parasitize more than one host species [41] or more rarely more than one genus [26], yet the possibility of them being cryptic species remains untested. More interestingly, in some cases more than one species can parasitize one host species [42] and sometimes they are specialized to different microhabitats such as the elytral cavity, on hindwings or on the dorsal abdomen of their host [39].

Australia is anticipated to harbour rich *Eutarsopolipus* fauna given its large diversity of carabid beetles [5]. This is inferred from small sampling efforts that have recently been conducted in some regions, and yet that discovered a considerable number of new species [31, 41–44]. Here, we describe three new species of *Eutarsopolipus* belonging to three different species groups (*leytei, myzus, pterostichi*) from three native Australian carabid beetles, raising the total number of Australian *Eutarsopolipus* to 30 species. All these species were recovered following a minimal sampling effort at one site, again corroborating the hypothesis that Australia is home to diverse podapolipid fauna awaiting discovery.

## Materials and methods

Carabid host beetles were collected at night on the ground, near an outdoor LED solar light lamp in Richmond, New South Wales, in February 2020. The subelytral area of the beetles (preserved in 75–80% ethanol) was subsequently examined for mite infestation. Mite specimens were cleared in a mixture of Nesbitt’s fluid and a small amount of glycerine slide mounted in Hoyer’s medium. Mite morphology was studied using a light microscope (Olympus BX51) equipped with phase contrast illumination. Mites from Queensland specimens of the carabid host *Gnathaphanus pulcher* were removed from dried beetles as described in Seeman [42] and examined using a Nikon 80i microscope equipped with differential interference contrast. All measurements are given in micrometres for holotypes and the range of measurements for five selected paratypes (in parentheses), if available. Distances between setae were measured from the base of one seta to the other; setae with their acetabulum remnant only were categorised as vestigial setae and those with their setae not extending past the acetabulum as microsetae (m). Terminology and setal notation were adapted from Lindquist [32]. The species group assignment follows that of Seeman [42]. Host beetles were all identified with the help of Geoff Monteith.

## Abbreviations

ap: apodem
appr: prosternal apodeme
apsej: sejugal apodeme
QM: Queensland Museum, QLD, Australia
ANIC: Australian National Insect Collection; Canberra, ACT, Australia
AC-DE-TMU: The Acarological Collection, Department of Entomology, Faculty of Agriculture, Tarbiat Modares University, Tehran, Iran

## Results

Family Podapolipidae Ewing, 1922

Genus *Eutarsopolipus* Berlese, 1913

Type species: *Tarsopolipus lagenaeformis* Berlese, 1911, by original designation.

Species group: *pterostichi* – Key characters of the group based on adult female: stigmata and tracheae absent; genua II–III without setae [42].

### *Eutarsopolipus paryavae* Katlav & Hajiqanbar n. sp. (Figs. 1–3)

urn:lsid:zoobank.org:act:36B8618D-FA09-474C-B4C3-2613DD962A5B

**Figure 1:**
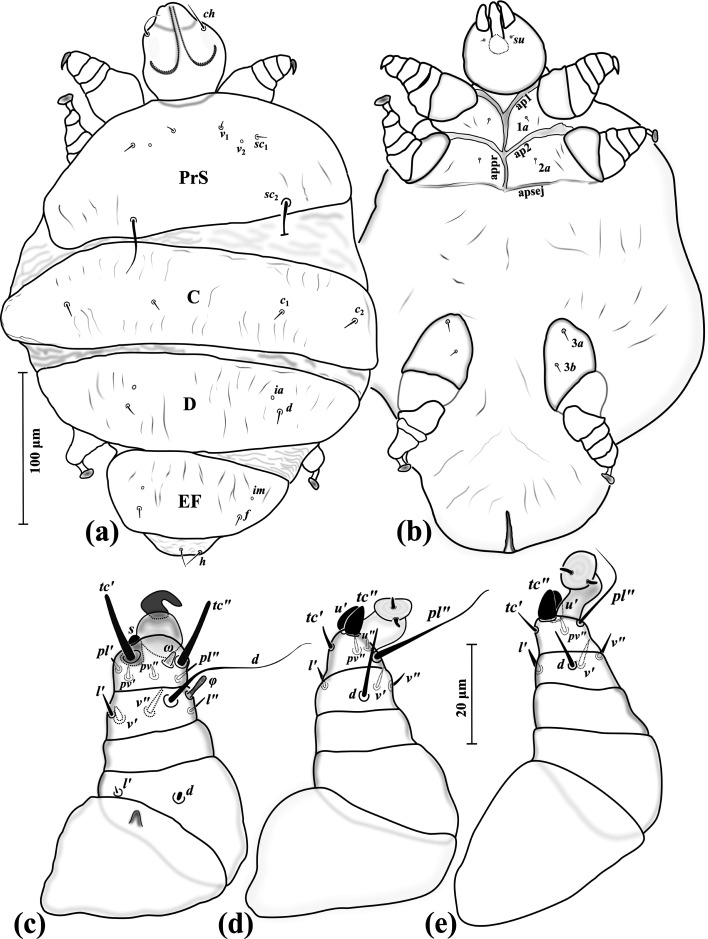
*Eutarsopolipus paryavae* n. sp. (adult female). (a) Body dorsum; (b) body venter; (c) right leg I; (d) ventral view of tarsus I; (e) right leg II; (f) right leg III. All legs in dorsal view.

**Figure 2:**
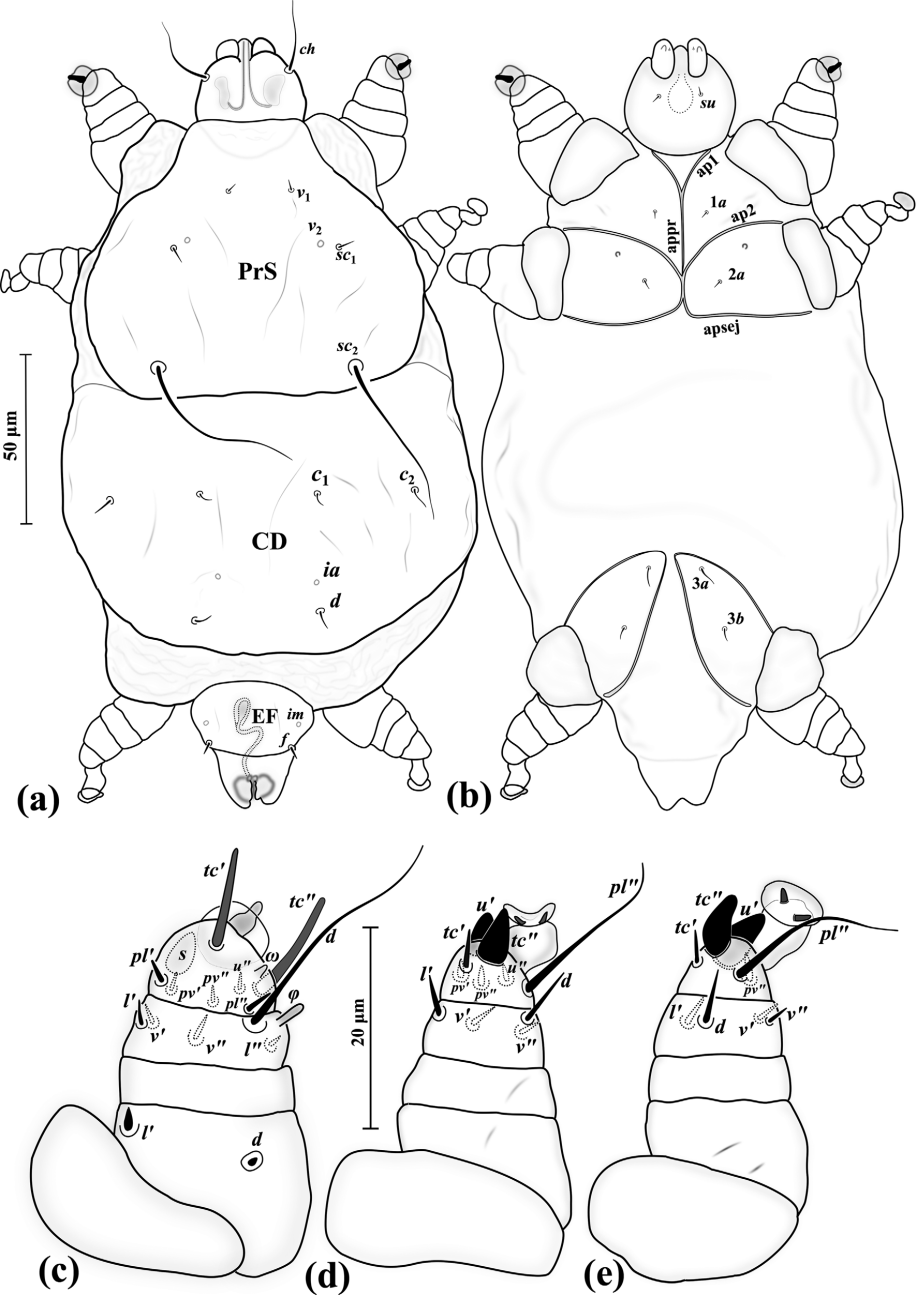
*Eutarsopolipus paryavae* n. sp. (male). (a) Body dorsum; (b) body venter; (c) right leg I; (d) right leg II; (e) right leg III. All legs in dorsal view.

**Figure 3:**
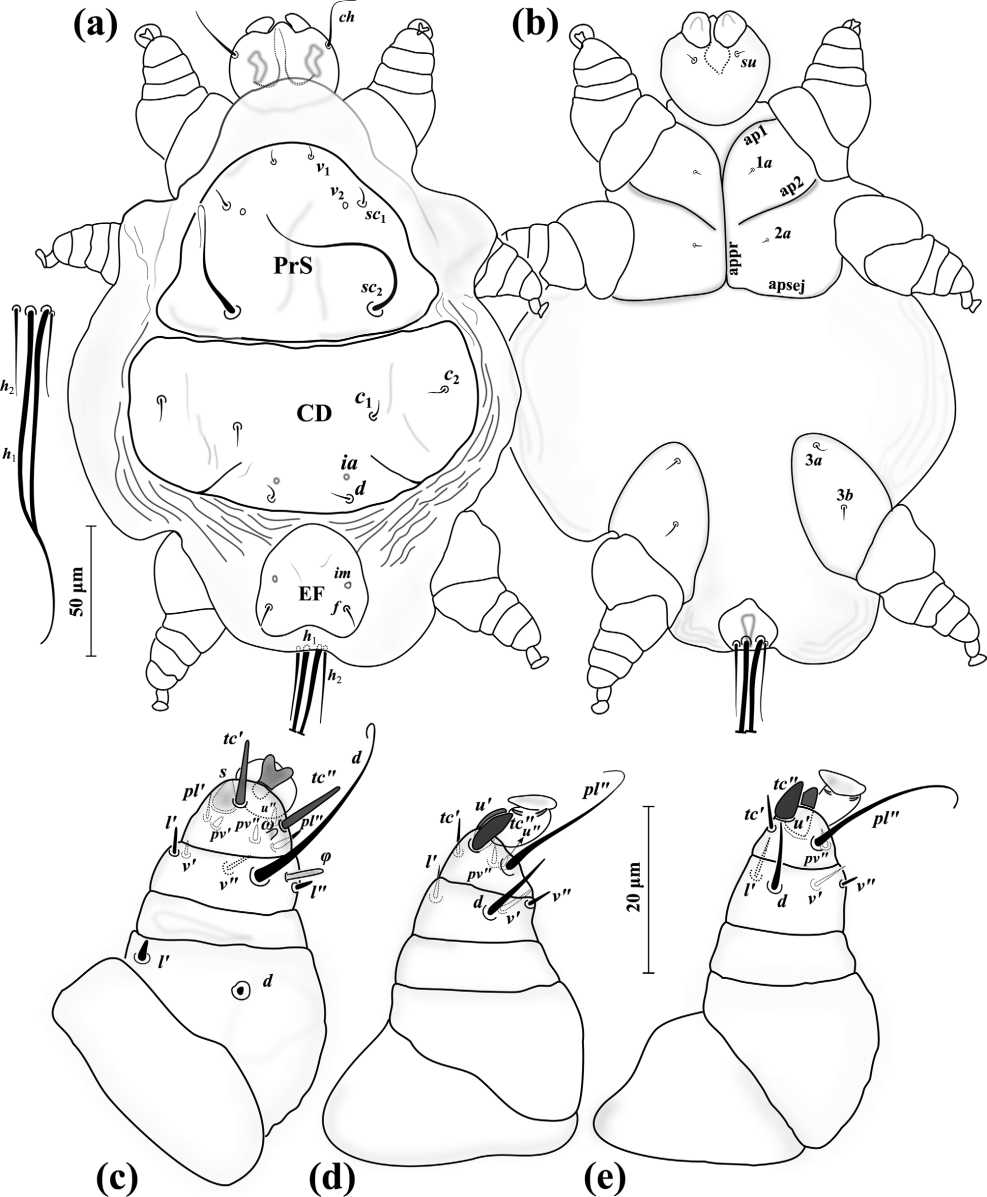
*Eutarsopolipus paryavae* n. sp. (larval female). (a) Body dorsum; (b) body venter; (c) right leg I; (d) right leg II; (e) right leg III. All legs in dorsal view.

*Type material*: *Total material recovered:* ♀ (*n* = 4), ♂ (*n* = 15), larval ♀ (*n* = 4), ex. under elytra, on the base of membranous hind wing of one specimen of *Geoscaptus laevissimus* Chaudoir, 1855 (Coleoptera: Carabidae: Scaritinae). *Holotype*: adult female (ANIC 52-003953), ex. under elytra, on the base of membranous hind wing of *G. laevissimus*; Coll. Shams Paryav; 11 Feb 2020. *Paratypes*: adult female (*n* = 3), male (*n* = 5) and larval female (*n* = 4), same data as holotype.

*Type locality*: Loc. Vines Drive, Hawkesbury Campus, Western Sydney University, Richmond, NSW, 33°36′45.6″ S 150°44′40.2″ E.

*Deposition of type material*: The holotype, one adult female, 2 male and 2 larval female paratypes are deposited at ANIC (ANIC 52-003953-58). 1 adult female, 2 males and 1 larval female paratypes are deposited at QM (QMS 117000-04). The remaining paratypes (TMU SP-20200211, 1–3), 10 non-type males and the host beetle specimen are deposited at AC-DE-TMU.

*Etymology:* The new species is named after the first author’s mother, Shams Paryav, the collector of the host beetle samples, in gratitude of her immense engagement in material collections.

*Authorship:* Note that the authors of the new taxon are different from the authors of this paper; Article 50.1 and Recommendation 50A of International Code of Zoological Nomenclature [24].

#### Description

##### Adult female (Fig. 1) (*n* = 4)

*Gnathosoma* (Figs. 1a–1b). Length 68 (72–86), width 58 (60–68); cheliceral stylets length 66 (63–68); pharynx length 14 (15–18), pharynx width 13 (13–14); *ch* 19 (21–26), *su* 3 (4–5); distance between setae *ch–ch* 34 (40–42), *su–su* 17 (19–22).

*Idiosoma* (Figs. 1a–1b). Length 300 (390–475), width 250 (295–335).

*Idiosomal dorsum* (Fig. 1a). All dorsal setae needle-like except attenuating setae *sc*_2_; prodorsal plate (PrS) with setae *v*_1_ 5 (4–5), *v*_2_ vestigial, *sc*_1_ 8 (7–8), *sc*_2_ 36 (35–38). Plate C setae *c*_1_ 8 (9–10), *c*_2_ 9 (9–11). Plate D setae *d* 9 (8–9); cupuli *ia* anterolaterad setae *d*. Plate EF setae *f* 7 (7–8); cupuli *im* anterolaterad setae *f*. Plate H not evident; setae *h* 12 (9–11). Distances between setae: *v*_1_–*v*_1_ 30 (31–35), *v*_2_–*v*_2_ 61 (62–69), *v*_1_–*v*_2_ 19 (21–25), *sc*_1_–*sc*_1_ 74 (76–87), *v*_1_–*sc*_1_ 27 (27–31), *sc*_2_–*sc*_2_ 103 (113–125), *sc*_1_–*sc*_2_ 49 (51–56), *c*_1_–*c*_1_ 85 (91–99), *c*_1_–*c*_2_ 49 (58–63), *d–d* 101 (104–107), *f–f* 67 (70–74), *h*_1_*–h*_1_ 11 (14–18).

*Idiosomal venter* (Fig. 1b). All coxal plates smooth; all coxal setae tiny needle-like; ap1–2 well developed, both reaching to appr; coxisternal field I with setae 1*a* 3 (3–4) and coxisternal field II with 2*a* 4 (3–4); alveoli of setae 1*b* and 2*b* not evident; coxisternal field III with setae 3*a* 7 (7–8) slightly longer than 3*b* 5 (5–6). Distances between setae: 1*a*–1*a* 25 (26–35), 2*a*–2*a* 30 (31–45), 3*a*–3*b* 21 (23–28).

*Legs* (Figs. 1c–1e). Setal formula for legs I–III (femur-tarsus): 2-0-5(+*φ*)-7(+*ω*), 0-0-4-6, 0-0-4-5. Ambulacrum I with well-developed sickle-shaped claw, ambulacrum II–III each with a pair of tiny claws. *Leg I* (Fig. 1c): femur, *d* microseta, slightly thickened, seta *l*′ 1 (m-1); tibia, *φ* 5 (6–7) clubbed, *d* 33 (29–35), *l*′ 4 (4–5), *l*″ 4 (4–6), *v*′ 3 (4–5) and *v*″ 5 (5–6) slightly thickened, seta *k* absent; tarsus, *ω* 3 (3–4) digitiform, eupathidial setae *tc*′ 14 (13–16) and *tc*″ 14 (14–15) distinctly blunt-ended, *pl*′ 3 (3–5), *pl*″ 5 (5-6), setae *pv*′ 2 (2–3) and *pv*″ 2 (2) slightly thickened, seta *s* 6 (6–7) blunt spur-like, *u*″ and *p*′ not evident. *Leg II* (Fig. 1d): tibia, *d* 14 (10–13), *l*′ 4 (4–5), *v*′ 5 (5–6), *v*″ 4 (4–4); tarsus, *tc*′ 5 (5–6), setae *u*′ 6 (7–8) and *tc*″ 6 (6–7) blunt spur-like, *pl*″ 28 (21–25), *pv*″ 4 (4–5), *u*″ 2 (2). *Leg III* (Fig. 1e): tibia, *d* 8 (7–9), *l*′ 5 (4–4), *v*′ 5 (5–6), *v*″ 5 (5); tarsus, *tc*′ 5 (5), setae *u*′ 6 (6–7) and *tc*″ 6 (6) blunt spur-like, *pl*″ 20 (22–24), *pv*″ 3 (4–4).

##### Male (Fig. 2) (*n* = 5)

*Gnathosoma* (Figs. 2a–2b). Length 33–36, width 32–33; cheliceral stylets length 23–26; pharynx length 9–10, pharynx width 7–8; *ch* 17–21, *su* 3–4; distance between setae *ch–ch* 25–26, *su–su* 12–14.

*Idiosoma* (Figs. 2a–2b). Length 145–210, width 120–130.

*Idiosomal dorsum* (Fig. 2a). All dorsal setae short (except *sc*_2_) and pointed; PrS with setae *v*_1_ 2–3, *v*_2_ vestigial, *sc*_1_ 4–6, setae *sc*_2_ 52–65 attenuate. Plate CD with seta *c*_1_ 4–5, *c*_2_ 6–7, *d* 5–6; cupuli *ia* anterior to setae *d*. Plate EF setae *f* 3–4; cupuli *im* anterolaterad setae *f*. Genital capsule length 31–34, width 25–30, situated posterior to margin of EF, setae *h*_1_ barely visible in few specimens. Distances between setae: *v*_1_–*v*_1_ 18–22, *v*_2_–*v*_2_ 40–43, *v*_1_–*v*_2_ 18–20, *sc*_1_–*sc*_1_ 50–55, *v*_1_–*sc*_1_ 20–22, *sc*_2_–*sc*_2_ 61–65, *sc*_1_–*sc*_2_ 37–41, *c*_1_–*c*_1_ 36–45, *c*_1_–*c*_2_ 29–40, *d–d* 38–40, *f–f* 21–25.

*Idiosomal venter* (Fig. 2b). All coxal plates smooth; all coxal setae pointed; ap1–2 and apsej well developed, all fused with appr; coxisternal field I with setae 1*a* 2, alveoli 1*b* not evident; coxisternal field II with 2*a* 3–3, alveoli 2*b* evident; coxisternal field III with setae 3*a* 5–6 slightly longer than 3*b* 4–4. Distances between setae: 1*a*–1*a* 15–19, 2*a*–2*a* 23–27, 3*a*–3*b* 19–20.

*Legs* (Figs. 2c–2e). Setal formula for legs I–III (femur-tarsus): 2-0-5(+*φ*)-8(+*ω*), 0-0-4-6, 0-0-4-5. Ambulacrum I with well-developed claw, ambulacrum II–III each with a pair of tiny claws. *Leg I* (Fig. 2c): femur, *d* microseta, slightly thickened, seta *l*′ 2–2 thickened; tibia, *φ* 4–5 clubbed, *d* 24–26, *l*′ 3, *l*″ 1–2, *v*′ 2–3, *v*″ 3–4, seta *k* absent; tarsus, *ω* 2 tiny, cone-shaped; eupathidial setae *tc*′ 10–12 and *tc*″ 11–12 distinctly blunt-ended, setae *pl*′ 3–4 and *pl*″ 3–4 slightly blunt-ended, *pv*′ 2, *pv*″ 2–2, seta *s* 4–5 blunt spur-like, *u*″ 1–2, seta *p*′ not evident. *Leg II.* (Fig. 2d): tibia, *d* 5–7, *l*′ 3–5, *v*′ 3–4, *v*″ 2–3; tarsus, seta *tc*′ 4–5, slightly blunt-ended; setae *u*′ 5–6 and *tc*″5–6 blunt spur-like, *pl*″ 19–20, *pv*″ 2–3, *u*″ 1–2. *Leg III* (Fig. 2e): tibia, *d* 5–6, *l*′ 3, *v*′ 3, *v*″ 3–3; tarsus, *tc*′ 3–4 slightly blunt-ended, setae *u*′ 6–7 and *tc*″ 5–6 blunt spur-like, *pl*″ 18–20, *pv*″ 3.

##### Larval female (Fig. 3) (*n* = 4)

*Gnathosoma* (Figs. 1a–1b). Length 35–39, width 41–42; cheliceral stylets length 30–33; pharynx length 10–12, pharynx width 9–11; *ch* 21–24, *su* 3–4; distance between setae *ch–ch* 35–38, *su–su* 16–17.

*Idiosoma* (Figs. 3a–3b). Length 220–235, width 135–175.

*Idiosomal dorsum* (Fig. 3a). All dorsal setae pointed; PrS with setae *v*_1_ 3–4, *v*_2_ vestigial, *sc*_1_ 6–7, *sc*_2_ 65–75. Plate C setae *c*_1_ 6–9, *c*_2_ 7–9. Plate D setae *d* 6–8; cupuli *ia* anterolaterad setae *d*. Plate EF setae *f* 7–8; cupuli *im* anterior to setae *f*. Plate H situated ventrally with setae *h*_1_ 130–140, *h*_2_ 29–32. Distances between setae: *v*_1_–*v*_1_ 13–16, *v*_2_–*v*_2_ 39–40, *v*_1_–*v*_2_ 20–23, *sc*_1_–*sc*_1_ 51–53, *v*_1_–*sc*_1_ 26–27, *sc*_2_–*sc*_2_ 53–55, *sc*_1_–*sc*_2_ 40–41, *c*_1_–*c*_1_ 49–53, *c*_1_–*c*_2_ 29–32, *d–d* 28–29, *f–f* 28–31.

*Idiosomal venter* (Fig. 3b). All coxal plates smooth; all coxal setae tiny needle-like; ap1 and apsej well developed, both fusing to appr; ap2 not reaching to appr; coxisternal field I with setae 1*a* 2–3, alveoli 1*b* not evident; coxisternal field II with 2*a* 2–3, alveoli 1*b* not evident; coxisternal field III with setae 3*a* 5–6 and 3*b* 5–6 subequal. Distances between setae: 1*a*–1*a* 17–22, 2*a*–2*a* 24–28, 3*a*–3*b* 24–27.

*Legs* (Figs. 3c–3e). Setal formula for legs I–III (femur-tarsus): 2-0-5(+*φ*)-8(+*ω*), 0-0-4-6, 0-0-4-5. Ambulacrum I with well-developed bifid claw with blunt tips, ambulacrum II–III each with a pair of barely discernible claws. *Leg I* (Fig. 3c): femur, *d* microseta, slightly thickened, seta *l*′ 2–3 slightly thickened; tibia, *φ* 5–5 baculiform, *d* 27–30, setae *l*′ 4 and *l*″ 2–3 slightly blunt-ended, *v*′ 2–3, *v*″ 4–5, seta *k* absent; tarsus, *ω* 2–2 cone-shaped with blunt tip, eupathidial setae *tc*′ 10–12 and *tc*″ 9 distinctly blunt-ended, *pl*′ 3–4, *pl*″ 4–5, setae *pv*′ 2–2 and *pv*″ 2–3 slightly thickened, seta *s* 5 blunt spur-like, *u*″ 2, seta not evident. *Leg II.* (Fig. 3d): tibia, *d* 10–12, *l*′ 4–5, *v*′ 4–5, *v*″ 3–4; tarsus, *tc*′ 5–5, setae *u*′ 5–7 and *tc*″ 5–7 blunt spur-like, *pl*″ 20–23, *pv*″ 2–3, *u*″ 2. *Leg III* (Fig. 3e): tibia, *d* 9–10, *l*′ 5–6, *v*′ 5, *v*″ 2–3; tarsus, *tc*′ 4–5, setae *u*′ 6–7 and *tc*″ 5–6 blunt spur-like, *pl*″ 20–23, *pv*″ 2–3.

#### Differential diagnosis

**Figure 4:**
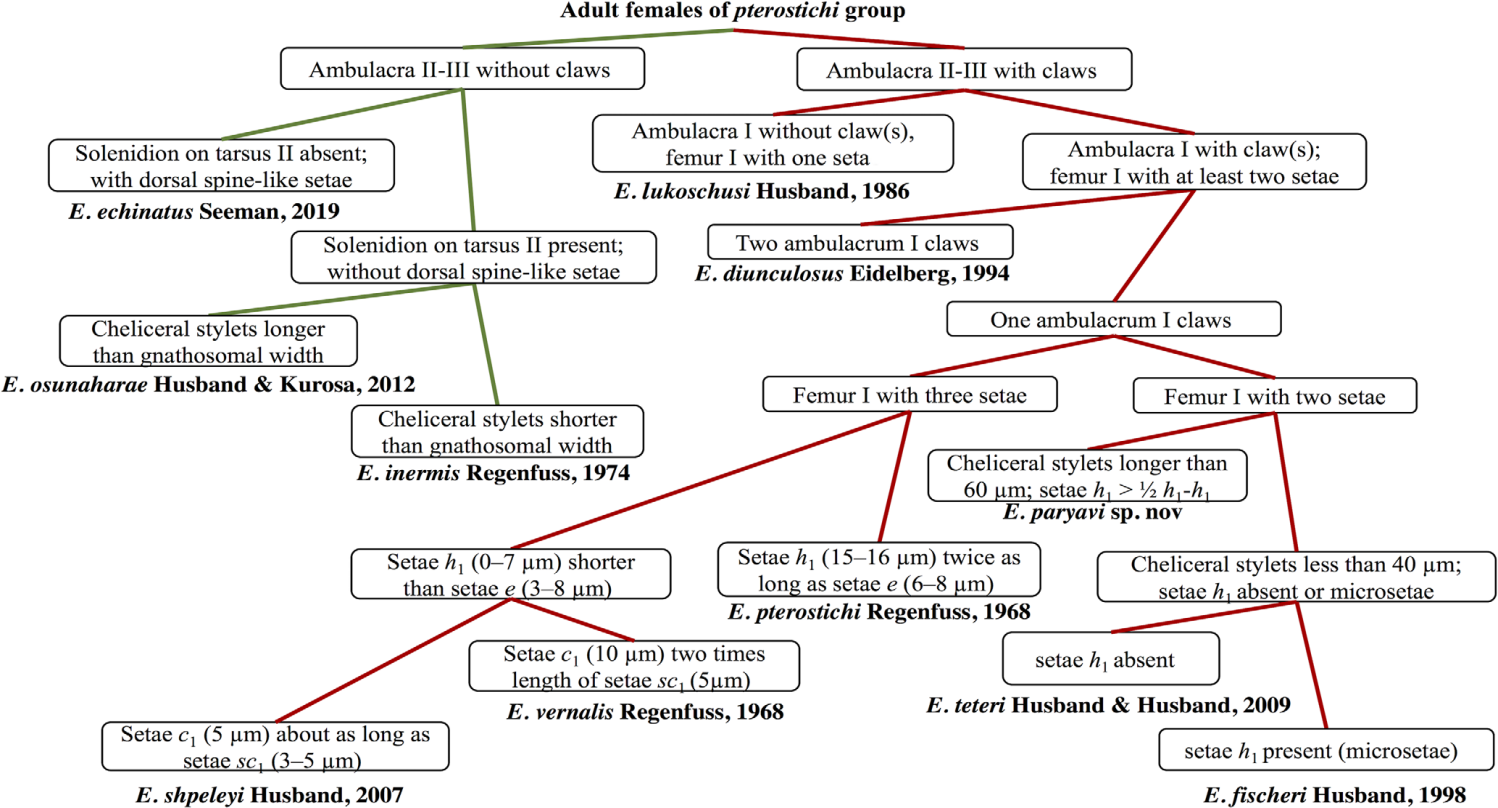
Key to the world species of *Eutarsopolipus* in the *pterostichi* group (based on adult females).

Within the *pterostichi* species group, the new species is most similar to *E. fischeri* Husband, 1998 and *E. teteri* Husband & Husband, 2009 in having ambulacra II and III with a pair of claws each and ambulacra I with one claw and femur I with two setae. However, it differs from both species in having cheliceral stylets longer than 60 (*vs.* shorter than 40 in both species), setae *h*_1_ 9–12 (absent in *E. teteri* and microsetae in *E. fischeri*) and seta *k* on tibia I absent (seta *k* on tibia I present in both species). The setal counts alone mask further differences. In *E. paryavae* and *E. fisheri*, the setae on femur I are the tiny setae *d* and *l*′, but in *E. teteri* seta *l*′ is absent and *v*′′ is present. Another important difference is the absence of a solenidion on tarsus II, which is present in *E. teteri* and probably present in *E. fischeri* (present in male and larva, absent or obscured in females). All the important characters among these three species are compared for all life stages in Table 1 and a key to the world species of the *pterostichi* group of *Eutarsopolipus* (based on adult females) is presented in Figure 4.

**Table 1:** Comparison of selected characters (range of measurements if available) of *E. paryavae* n. sp. (*Ep*) with two closely related species of the pterostichi species group: *E. fischeri* (*Ef*) and *E. teteri* (*Et*). Dashes (–) denote absence of the character. Characters for which the data are obscured/not available in the original descriptions are given as question marks (?). Letters v and m indicate vestigial and mirosetae, respectively. Abbreviations: L. (length), S. (stylet), Gn. (gnathosoma), Gen. cap. (genital capsule), Ch. (chelicera), Sol. (solenidion), Ta (tarsus), Ti (tibia), Fe (femur).

Life stage	Female			Male			Larval female		
Character	*Ep*	*Ef*	*Et*	*Ep*	*Ef*	*Et*	*Ep*	*Ef*	*Et*
Gn. L.	68–86	45–48	45	33–36	26–29	29–32	35–39	31	35–38
Ch. S. L.	63–68	33–34	38	23–26	19–22	22	30–33	22	32–34
Setae *ch*	19–26	17–19	15	17–21	2–4	5	21–24	17	20–25
Setae *su*	3–5	5–6	14	3–4	2–3	8	3–4	3	10–12
Setae *v*_1_	4–5	5–6	10	2–3	2	?	3–44	5	15
Setae *sc*_1_	7–8	6–7	9	4–6	2	?	6–7	3	9–10
Setae *sc*_2_	35–38	32–42	59	52–65	48	38–45	65–75	74	80
Setae *c*_1_	8–10	3–5	5	4–5	2	?	7–9	3	8–12
Setae *c*_2_	9–11	5–6	7	6–7	2	?	7–9	4	10
Setae *d*	8–9	3–5	7	5–6	?	?	6–8	4	10
Setae *f*	7–8	5	5	3–4	?	?	7–8	4	7–10
Setae *h*_1_	9–12	m	–	–	–	–	130–140	43	60–62
Setae *h*_2_	–	–	–	–	–	–	29–32	16	5–7
Setae 1*a*	3–4	2	m	2	v	m–3	2–3	m	4
Setae 2*a*	3–4	2–3	7	3	v	3	2–3	m	5–6
Setae 3*a*	7–8	4–5	?	5–6	v	?	5–6	3	?
Setae 3*b*	5–6	7	3	4	?	m	5–6	4	5
Gen. cap. L.	–	–	–	31–34	25–38	38	–	–	–
Gen. cap. W.	–	–	–	25–30	27–30	32	–	–	–
Sol. Ta I *ω*	3–4	3	5	2	2–3	5	2	3	4–5
Sol. Ti I *φ*	5–7	7–10	7	4–5	7–8	5–7	4–5	6	8–13
Sol. Ta II *ω*	–	?	5	–	2–3	5	–	3	5
Fe I seta *v″*	–	–	15	–	–	10–18	–	–	10
Fe I seta *l*′	m–1	~3–4	–	2	~1	–	2–3	~2	–
Ta III seta *pl*″	20–24	15–17	15	18–20	12	10–15	20–23	13	15–18

Species group: *leytei* – Key characters of the group based on adult females: stigmata and tracheae present; ambulacral claws II–III present; genu II–III with setae [42].

### *Eutarsopolipus pulcher* Hajiqanbar & Seeman n. sp. (Figs. 5–8)

urn:lsid:zoobank.org:act:33189535-1D7E-4118-B46D-7EA9AE8E0207

**Figure 5:**
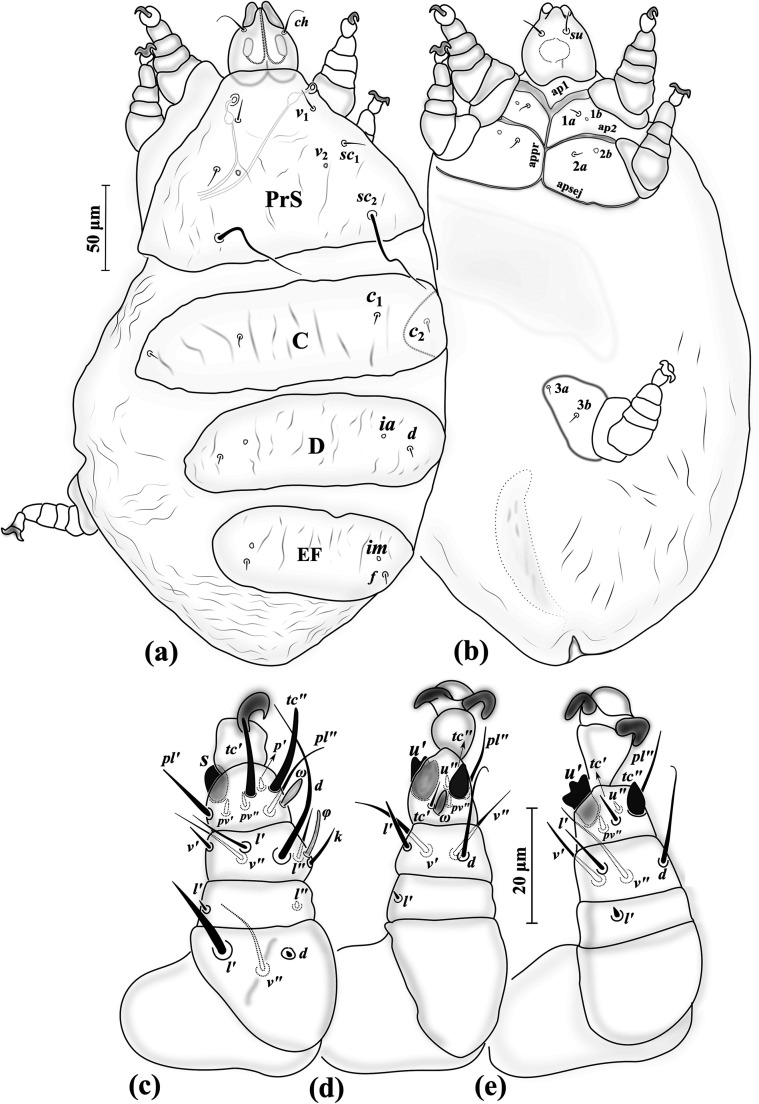
*Eutarsopolipus pulcher* n. sp. (adult female). (a) Body dorsum; (b) body venter; (c) right leg I; (d) ventral view of tarsus I; (e) right leg II; (f) right leg III. All legs in dorsal view.

**Figure 6:**
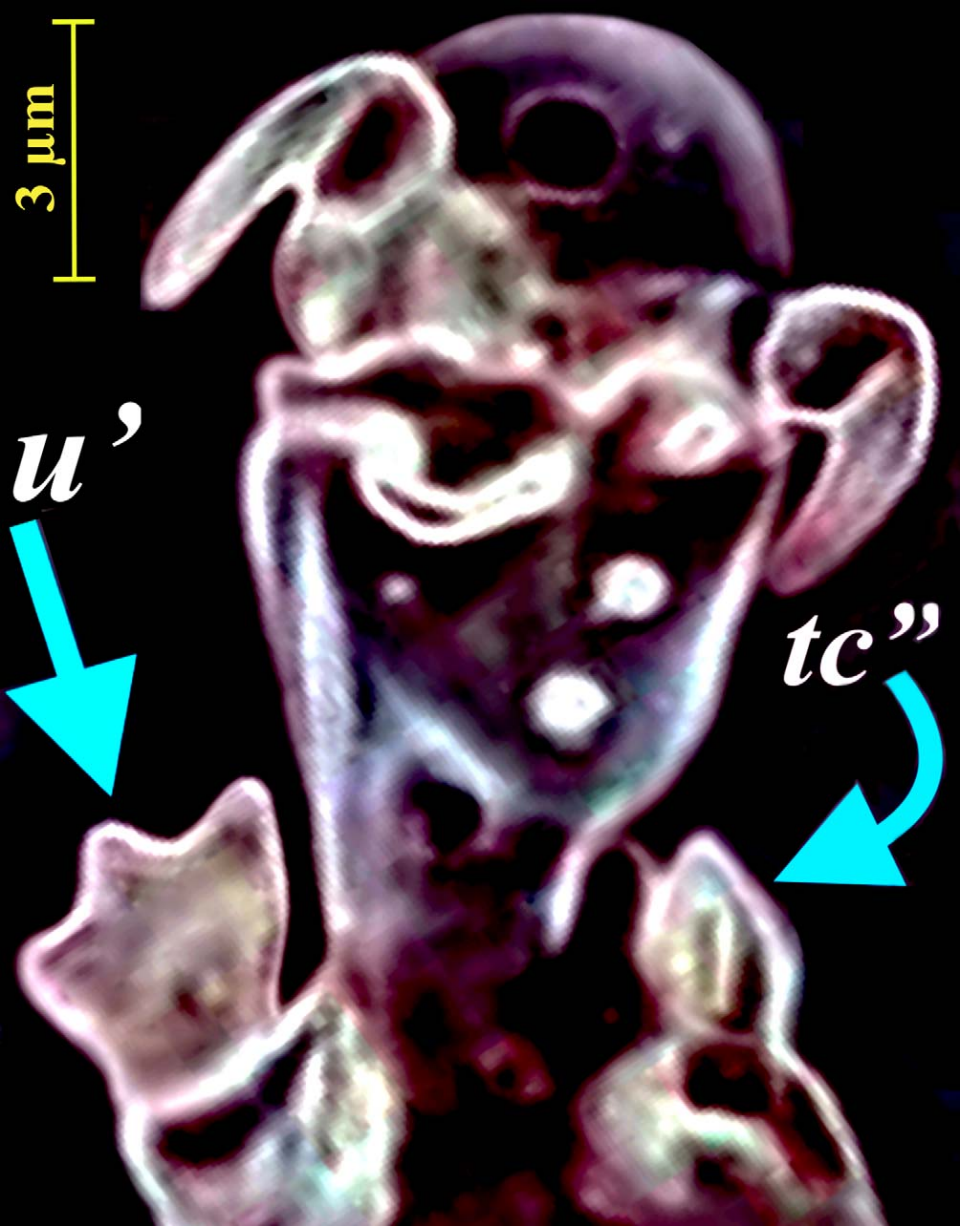
Phase-contrast micrograph of tarsus III in *Eutarsopolipus pulcher* n. sp. (adult female) representing modified trifurcate seta *u′* and spur-like seta *tc″*.

**Figure 7:**
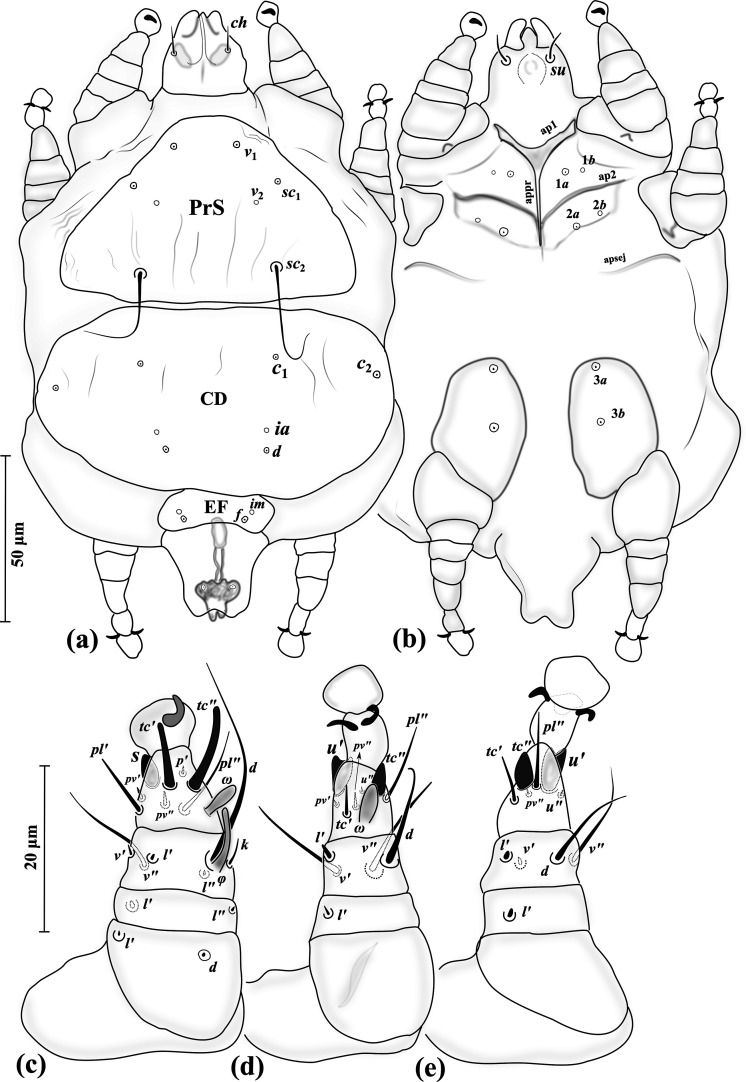
*Eutarsopolipus pulcher* n. sp. (male). (a) Body dorsum; (b) body venter; (c) right leg I; (d) right leg II; (e) right leg III. All legs in dorsal view.

**Figure 8:**
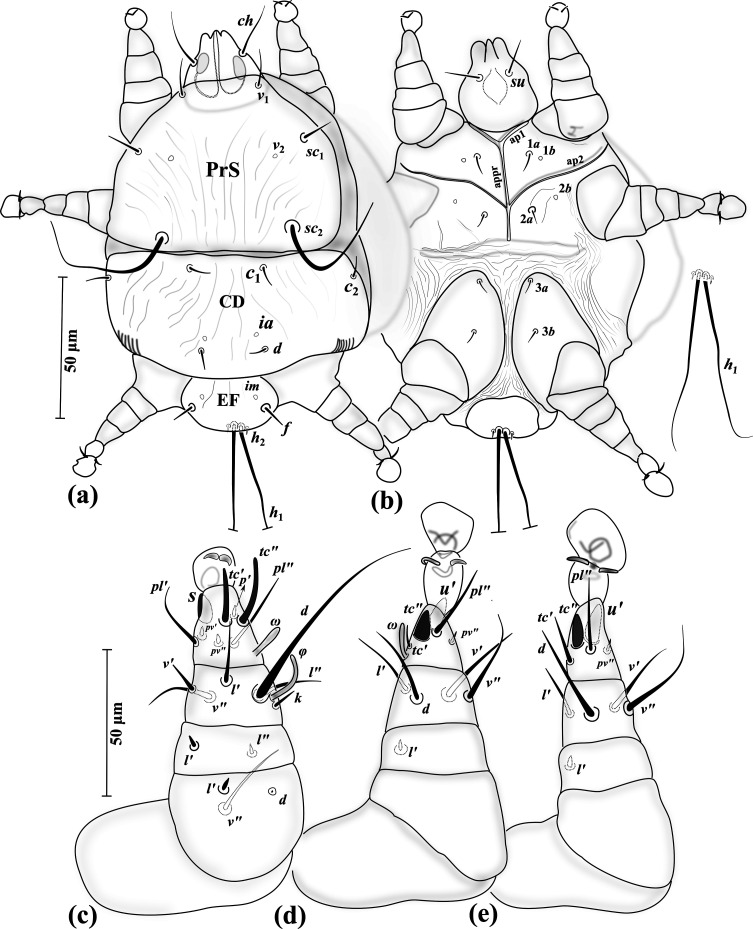
*Eutarsopolipus pulcher* n. sp. (larval female). (a) Body dorsum; (b) body venter; (c) right leg I; (d) right leg II; (e) right leg III. All legs in dorsal view.

*Type material. Total material recovered*: adult female (*n* = 12), male (*n* = 6), larval female (*n* = 13), ex. under elytra, on the base of membranous hind wing of specimens of *Gnathaphanus pulcher* (Dejean, 1829) (Coleoptera: Carabidae; Harpalinae). Four out of ca. 160 examined host specimens found parasitized (4% prevalence). Samples were collected at four independent events on 14 Feb 2020, 26 Feb 2020, 30 Feb 2020 and 3 March 2020. *Holotype*: adult female (ANIC 52-003959), ex. under elytra, on the base of membranous hind wing of *G. pulcher*; Coll. Shams Paryav; 14 Feb 2020. *Paratypes*: adult female (*n* = 5), male (*n* = 5), larval female (*n* = 5), same data as holotype.

*Type locality*: Loc. Vines Drive, Hawkesbury Campus, Western Sydney University, Richmond, NSW, 33°36′45.6″ S 150°44′40.2″ E.

*Deposition of types*: The holotype, one adult female, 2 male and 2 larval female paratypes are deposited at ANIC (ANIC 52-003959-54). 2 adult female, 2 males and 1 larval female paratypes are deposited at QM (QMS 117005-10). The remaining paratypes (TMU SP-20200214, 1–3) and the host beetle specimen are deposited at AC-DE-TMU.

*Other material examined*: adult female (*n* = 21), male (*n* = 4), larval female (*n* = 3), ex. under elytra, on the base of membranous hind wing of *G. pulcher* (host registration number T137238), Loc. “Feez Creek” property entrance, QLD, 21°51′40″ S 148°14′19″ E; Coll. S. Wright & C. Burwell; 9 Mar 2005 (QMS 117011-38). Adult female (*n* = 1), larval female (*n* = 2), same data except different beetle (host registration number T137239) (QMS 117039-41).

*Etymology*: The new species name “*pulcher*” is adopted after the species name of the carabid host beetle *G. pulcher* meaning “beautiful” in Latin that is associated with the beautiful metallic colouration patterns of elytra in this beetle. Furthermore, this epithet has a proper relevance to the beautiful trifurcate setae *u*′ on tarsi II–III in adult females of the new mite species.

*Authorship*: Note that the authors of the new taxon are different from the authors of this paper; Article 50.1 and Recommendation 50A of International Code of Zoological Nomenclature [24].

#### Description

##### Adult female (Fig. 5) (*n* = 6)

*Gnathosoma* (Figs. 5a–5b). Length 45 (42–50), width 40 (34–41); cheliceral stylets length 51 (44–48); pharynx length 18 (16–18), pharynx width 14 (13–16); *ch* 18 (19–25), *su* 13 (12–14); distance between setae *ch–ch* 24 (23–26), *su–su* 14 (14–16).

*Idiosoma* (Figs. 5a–5b). Length 350 (270–415), width 215 (180–290).

*Idiosomal dorsum* (Fig. 5a). All dorsal setae pointed; prodorsal plate (PrS) with setae *v*_1_ 13 (11–14), *v*_2_ vestigial, *sc*_1_ 11 (9–13), *sc*_2_ 57 (51–60). Plate C setae *c*_1_ 6 (4–6), *c*_2_ 6 (4–6). Plate D setae *d* 5 (4–5); cupuli *ia* evident, anterolaterad setae *d*. Plate EF setae *f* 5 (4–5); cupuli *im* evident, anterolaterad setae *f*. Plate H and setae *h*_1_ not evident. Distances between setae: *v*_1_–*v*_1_ 46 (46–49), *v*_2_–*v*_2_ 51 (49–59), *v*_1_–*v*_2_ 34 (32–34), *sc*_1_–*sc*_1_ 76 (74–80), *v*_1_–*sc*_1_ 27 (27–29), *sc*_2_–*sc*_2_ 93 (88–99), *sc*_1_–*sc*_2_ 70 (68–71), *c*_1_–*c*_1_ 81 (75–92), *c*_1_–*c*_2_ 54 (49–54), *d–d* 113 (102–112), *f–f* 83 (73–93).

*Idiosomal venter* (Fig. 5b). All coxal plates smooth; all coxisternal setae tiny needle-like; ap1–2 and apsej well developed, reaching to appr; coxisternal field I with setae 1*a* 5 (5–6); alveoli of vestigial setae 1*b* evident; coxisternal field II with 2*a* 5 (5–7); alveoli of vestigial setae 2*b* evident; coxisternal field III with setae 3*a* 4 (4–5) and 3*b* 6 (5–6). Distances between setae: 1*a*–1*a* 31 (29–35), 2*a*–2*a* 34 (36–42), 3*a*–3*b* 26 (22–27).

*Legs* (Figs. 5c–5e, 6). Setal formula for legs I–III (femur-tarsus): 3-2-6(+*φ*)-8(+*ω*), 0-1-4-6 (+*ω*), 0-1-4-6. Ambulacrum I with sickle-shaped claw, ambulacrum II–III each with a pair of well-developed claws. *Leg I* (Fig. 5c): femur, *d* microseta, seta *l*′ 15 (14–16) pointed and thickened, subequal to *v*″ 15 (12–15); genu with tiny setae *l*′ 2 (2–2) and *l*″ 1 (1–2); tibia with *φ* 8 (7–9) baculiform, *d* 29 (25–29), *l*′ 11 (9–11), *l*″ 9 (7–9), *v*′ 5 (5–6) stiff, *v*″ 14 (13–16), seta *k* 8 (8–10); tarsus I, *ω* 5 (4–5) digitiform, eupathidial setae *tc*′ 14 (12–15) and *tc*″ 15 (13–15) distinctly blunt-ended, *pl*′ 11 (11–13), *pl*″ 15 (13–17), setae *pv*′ 3 (3–3) and *pv*″ 2 (2–3) subequal, seta *s* 6 (6–7) modified and thickened, *p*′ 2 (2) slightly thickened. *Leg II.* (Fig. 5d): genu, *l*′ 2 (2); tibia, *d* 17 (15–17), *l*′ 9 (7–9), *v*′ 14 (12–14), *v*″ 15 (13–19); tarsus II, *ω* 4 (3–4) digitiform, *tc*′ 5 (5–7), setae *u*′ 8 (7–8) spine-like and trifurcate, *tc*″ 6 (6–7) blunt spur-like, *pl*″ 13 (12–13), *pv*″ 2 (2–3), *u*″ 2 (2). *Leg III* (Figs. 5e, 6): genu, *l*′ 2 (2–2); tibia, *d* 17 (15–18), *l*′ 9 (7–9), *v*′ 14 (13–14), *v*″ 17 (15–18); tarsus III, *tc*′ 5 (5–7), setae *u*′ 8 (7–8) spine-like and trifurcate (Fig. 6), *tc*″ 6 (5–6) blunt spur-like (Fig. 5f), *pl*″ 14 (12–14), *pv*″ 2 (2–3), *u*″ 14 (12–14).

##### Male (Fig. 7) (*n* = 5)

*Gnathosoma* (Figs. 7a–7b). Length 25–36, width 23–27; cheliceral stylets length 17–19; pharynx length 9–10, pharynx width 6–8; *ch* 8–12; *su* 9–10; distance between setae *ch–ch* 17–20, *su–su* 12–13.

*Idiosoma* (Figs. 7a–7b). Length 140–160, width 105–115.

*Idiosomal dorsum* (Fig. 7a). All setae on dorsum microsetae (except *sc*_2_); PrS with setae *v*_2_ vestigial, setae *sc*_2_ 34–46 attenuate and pointed. Plate CD with cupuli *ia* anterior to setae *d*. Plate EF setae with cupuli *im* anterolaterad setae *f*. Genital capsule length 23–30, width 28–33, situated posterior to margin of EF, setae *h*_1_ barely visible on genital capsule. Distances between setae: *v*_1_–*v*_1_ 18–19, *v*_2_–*v*_2_ 30–33, *v*_1_–*v*_2_ 18–19, *sc*_1_–*sc*_1_ 44–47, *v*_1_–*sc*_1_ 17–18, *sc*_2_–*sc*_2_ 42–45, *sc*_1_–*sc*_2_ 26–28, *c*_1_–*c*_1_ 39–43, *c*_1_–*c*_2_ 25–29, *d–d* 29–34, *f–f* 19–21.

*Idiosomal venter* (Fig. 7b). All coxal plates smooth; all ventral setae on coxal area microsetae; ap1-2 well developed, fused with appr, apsej weekly developed, not reaching appr; alveoli of setae 1*b* on coxisternal field I evident; on coxisternal field II alveoli of setae 2*b* evident. Distances between setae: 1*a*–1*a* 17–19, 2*a*–2*a* 22–24, 3*a*–3*b* 18–20.

*Legs* (Figs. 7c–7e). Setal formula for legs I–III (femur-tarsus): 2-2-6(+*φ*)-8(+*ω*), 0-1-4-6(+*ω*), 0-1-4-6. Ambulacrum I with a small claw, ambulacrum II–III each with a pair of small claws. *Leg I* (Fig. 7c): femur, setae *d* and *l*′ microsetae; genu, setae *l*′ and *l*″ microsetae; tibia, *φ* 6–8 baculiform, *d* 21–25, *l*′ and *l*″ microsetae, *v*′ 1, *v*″ 12–14, seta *k* 3–5; tarsus, *ω* 4–5 digitiform; eupathidial setae *tc*′ 8–10 and *tc*″ 10–11 distinctly blunt-ended, setae *pl*′ 8–9 and *pl*″ 10–12, seta *pv*′ 1–1 stiff and blunt-ended, *pv*″ 2–2, seta *s* 4–5 blunt spur-like, *p*′ 1–1. *Leg II.* (Fig. 7d): genu, *l*′ 1; tibia, *l*′ 2, *d* 13–15, *v*′ 11–14, *v*″ 11–14; tarsus, *ω* 4–5 thickened and digitiform, seta *tc*′ 4–5, slightly blunt-ended, *u*′ 4–6 spine-like and bifurcate, *tc*″ 4–5 blunt spur-like, *pl*″ 9–11, *pv*′ 2, *pv*″ 2, *u*″ 1. *Leg III* (Fig. 7e): genu, *l*′ 1; tibia, *d* 10–13, *l*′ 1, *v*′ 1, *v*″ 12–14; tarsus, *tc*′ 5–7 stiff and slightly blunt-ended, setae *u*′ 5–6 spine-like and bifurcate, *tc*″ 4–5 blunt spur-like, *pl*″ 9–10, *pv*″ 1, *u*″ 1.

##### Larval female (Fig. 8) (*n* = 5)

*Gnathosoma* (Figs. 8a–8b). Length 29–32, width 24–29; cheliceral stylets length 28–34; pharynx length 10–13, pharynx width 7–9; *ch* 20–25; *su* 10–12; distance between setae *ch–ch* 15–18, *su–su* 10–11.

*Idiosoma* (Figs. 8a–8b). Length 125–145, width 95–110.

*Idiosomal dorsum* (Fig. 8a). All dorsal setae needle-like except *sc*_2_ which is long and attenuate; PrS with setae *v*_1_ 11–13, *v*_2_ vestigial, *sc*_1_ 10–12, *sc*_2_ 62–72. Plate C setae *c*_1_ 7–8, *c*_2_ 5–7. Plate D setae *d* 6–7; cupuli *ia* anterolaterad setae *d*. Plate EF setae *f* 7–9; cupuli *im* anterior to setae *f*. Plate H not evident; setae *h*_1_ 64–66, *h*_2_ m–2. Distances between setae: *v*_1_–*v*_1_ 25–28, *v*_2_–*v*_2_ 35–37, *v*_1_–*v*_2_ 22–25, *sc*_1_–*sc*_1_ 56–60, *v*_1_–*sc*_1_ 23–26, *sc*_2_–*sc*_2_ 44–48, *sc*_1_–*sc*_2_ 30–32, *c*_1_–*c*_1_ 25–28, *c*_1_–*c*_2_ 31–34, *d–d* 22–25, *f–f* 25–30.

*Idiosomal venter* (Fig. 8b). All coxal plates smooth; all coxal setae tiny needle-like; ap1–2 well developed, both fusing to appr; apsej not evident; coxisternal field I with setae 1*a* 5–7; alveoli of setae 1*b* on coxisternal field I evident; coxisternal field II with 2*a* 4–6; alveoli of setae 2*b* evident; coxisternal field III with setae 3*a* 5–6 and 3*b* 5 subequal. Distances between setae: 1*a*–1*a* 17–21, 2*a*–2*a* 16–22, 3*a*–3*b* 17–19.

*Legs* (Figs. 8c–8e). Setal formula for legs I–III (femur-tarsus): 3-2-6(+*φ*)-8(+*ω*), 0-1-4-5(+*ω*), 0-1-4-5. Ambulacrum I with a small bifid claw, ambulacrum II–III each with a pair of small claws. *Leg I* (Fig. 8c): femur, *d* microseta, seta *l*′ 2 slightly thickened, *v*″ 10–11; genu, *l*′ 2, *l*″ 1–1; tibia, *φ* 7–8 baculiform and bent, *d* 28–30, setae *l*′ 8–9 and *l*″ 9–12, *v*′ 4–5, *v*″ 10–12, seta *k* 3–4; tarsus, *ω* 4–5 digitiform, eupathidial setae *tc*′ 8–9 and *tc*″ 9–10 distinctly blunt-ended, *pl*′ 9–10, *pl*″ 12–14, setae *pv*′ 1–1 and *pv*″ 1–2, seta *s* 3–5 blunt spur-like, *p*′ 2. *Leg II.* (Fig. 8d): genu, *l*′ 2; tibia, *d* 13–18, *l*′ 10–11, *v*′ 10–13, *v*″ 13–15; tarsus, *ω* 4–5 digitiform, *tc*′ 3–4, setae *u*′ 5 and *tc*″ 4–5 blunt spur-like, *pl*″ 9–11, *pv*″ 2, *u*″ not evident. *Leg III* (Fig. 8e): genu, *l*′ 2; tibia, *d* 14–15, *l*′ 8–12, *v*′ 7–11, *v*″ 11–12; tarsus, *tc*′ 6–7, setae *u*′ 5–6 and *tc*″ 5–6 blunt spur-like, *pl*″ 10–11, *pv*″ 2, *u*″ not evident.

#### Differential diagnosis

This new species is unique in *Eutarsopolipus* by having trifurcate setae *u*′ on tarsi II–III. However, among species with simple claws on legs I (unlike *E. biuncatus* Seeman, 2021 and *E. janus* Seeman, 2021 with bifurcate claws on legs I), it is most similar to *E. leytei* Husband & Raros, 1989 with femur I seta *lʹ* very short, not reaching genual base in adult females; but it is readily distinguishable from this species by longer setae *v*_1_ 11–14 (*m–5* in *E. leytei*) and shorter cheliceral stylets being at most 51 in *E. pulcher* n. sp. vs. 68 in *E. leytei*.

**Figure 9:**
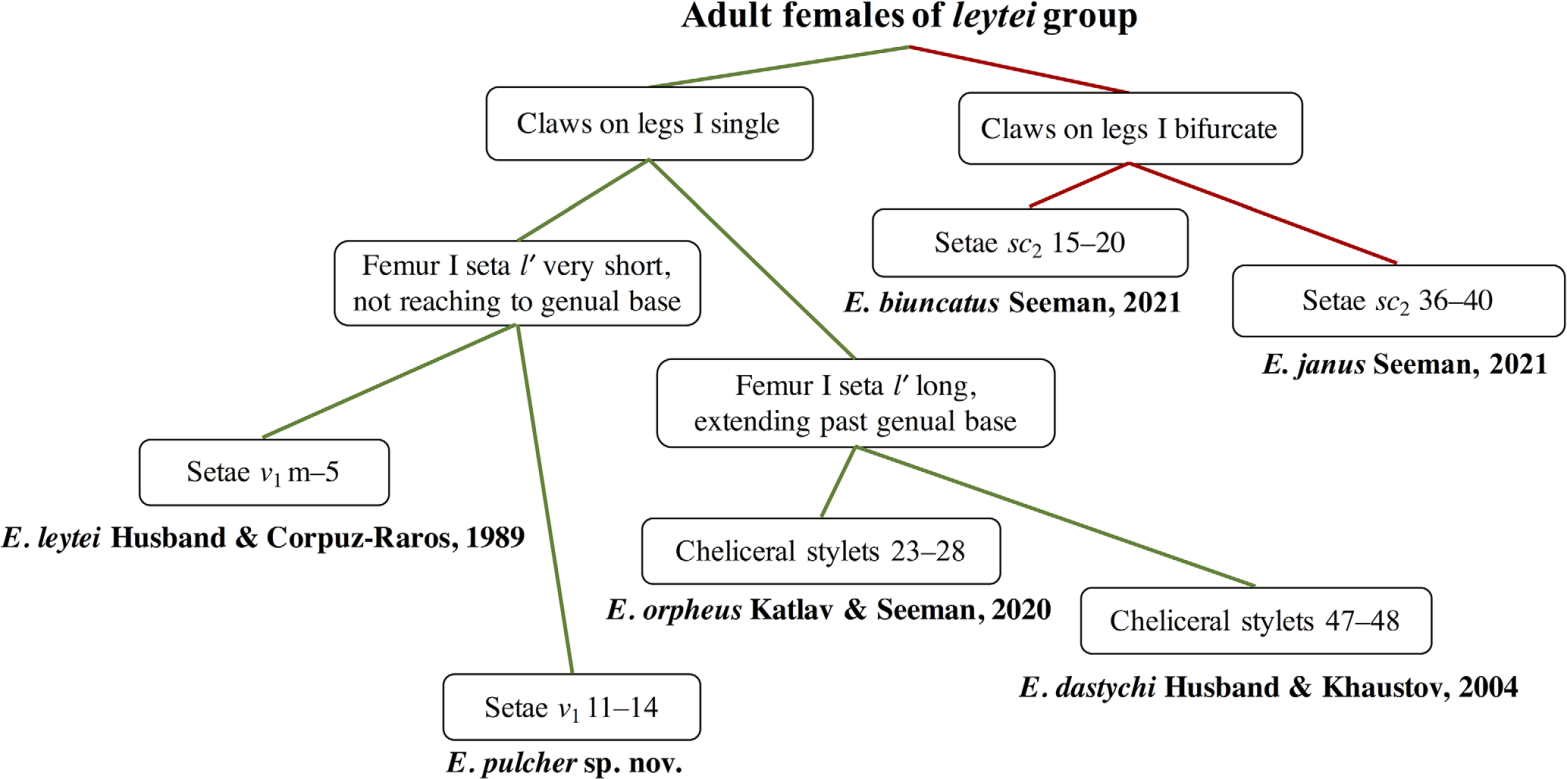
Key to the world species of *Eutarsopolipus* in the *leytei* group (based on adult females).

The new species further differs from *E. dastychi* with setae *v*_1_ longer than *ch* and setae *c*_1_, *c*_2_, *d* and *f* shorter than 8 in adult females (*vs.* setae *v*_1_ shorter than *ch* and setae *c*_1_, *c*_2_, *d* and *f* longer than 15 in adult females of *E. dastychi*). The male of *E. pulcher* n. sp. resembles that of *E. orpheus* with all ventral and dorsal setae (except *sc*_2_) being microsetae, but it differs from *E. orpheus* with setae *ch* longer than 8 (*ch* microsetae in male of *E. orpheus*). The larval female of *E. pulcher* n. sp. is similar to *E. orpheus* with *h*_1_ shorter than 70 and *h*_2_ shorter than 2, but it is readily distinguishable from *E. pulcher* n. sp. by shorter setae *sc*_1_, *sc*_2_, *c*_1_, *c*_2_, *d,* 3*a* and 3*b* (Table 2). All the important characters among the species of *leytei* group are compared for all life stages (excluding *E. leytei* with unknown male) in Table 2 and keys to the world species (based on adult females) are presented in Figure 9.

**Table 2:** Comparison of selected characters (range of measurements if available) of all the described species of the *leytei* species group in the genus *Eutarsopolipus* (male is unknown for *E. leytei*); species abbreviated as *E. pulcher* n. sp. (*Ep*), *E. leytei* (*El*), *E. dastychi* (*Ed*), *E. orpheus* (*Eo*), *E. biuncatus* (*Eb*), and *E. janus* (*Ej*).

Life stage	Female						Male					Larval female					
Character	*Ep*	*El*	*Ed*	*Eo*	*Eb*	*Ej*	*Ep*	*Ed*	*Eo*	*Eb*	*Ej*	*Ep*	*El*	*Ed*	*Eo*	*Eb*	*Ej*
Gn. L.	42–50	78	47–50	50–57	44–46	52–57	25–36	30–33	30–32	28–31	28–37	29–32	56	37–40	32–38	30–34	31–34
Ch. S. L.	44–51	68	47–48	23–28	35–38	45–51	17–19	23–26	15	17–18	25–28	28–34	54	35–40	21–27	25–27	36–42
Setae *ch*	18–25	20	27–30	10–18	14–22	25–35	8–12	12–15	m	1–2	3–4	20–25	20	30–36	31–35	19–23	26–33
Setae *su*	12–14	3	22	11–15	8–12	15–20	9–10	9–10	6–7	4–6	8–9	10–12	3	15–18	6–7	8–10	12–15
Setae *v*_1_	11–14	5	36–45	17–26	8–10	19–24	m	5–10	m	m–2	4–9	11–13	m	42–50	26–32	12–14	15–18
Setae *sc*_1_	9–13	11	25	17–29	7–9	9–11	m	5–13	m	m	m–2	10–12	10	25–28	28–33	5–6	8–10
Setae *sc*_2_	51–60	62	58–70	47–59	15–20	36–40	34–46	60–70	38–40	2–3	27–34	62–72	78	94–101	90–95	51–52	70–80
Setae *c*_1_	4–6	12	18–19	8–10	5–7	8–9	m	10	m	m	2–3	7–8	10	18–22	14–18	5–6	7–10
Setae *c*_2_	4–6	9	17–21	9–18	4–6	8–10	m	5–7	m	m	2	5–7	9	17–21	10–15	3–4	7–10
Setae *d*	4–5	12	18–21	11–18	4–5	9–10	m	8–10	m	m	m–2	6–7	9	20–23	15–17	4–5	7–9
Setae *f*	5	8	22	10–11	4–5	7–9	m	3–4	m	m	m–2	7–9	8	16–18	8–12	4–5	6–8
Setae *h*_1_	–	–	–	–	–	–	m	m	m	m	m	64–67	148	65–90	55–64	65–70	100
Setae *h*_2_	–	–	–	–	–	–	–	–	–	–	–	1–2	67	m	m	3–4	4–6
Setae 1*a*	5–6	~4	5–6	4–5	5–6	6–7	m	2–5	m	2–3	4–5	5–7	~2	8–10	6–8	2–3	4–5
Setae 2*a*	5–7	~4	5	3–4	4–5	6–7	m	3–4	m	2–3	4–5	4–6	~2	7–10	8–9	3–4	4–5
Setae 3*a*	4–5	8	9	2–3	5–6	6–9	m	~2	m	3	5–6	5–6	4	10–13	9–11	4–5	7–8
Setae 3*b*	5–6	4	7	3	4–5	6–7	m	~4	m	2–3	5–6	5	2	10–12	9–10	3–4	5–6
Sol. Ta I *ω*	4–5	~2	4–5	5–6	3–4	4–5	4–5	5–7	4	3	3–4	4–5	~2	4–5	4–5	3–4	4–5
Sol. Ti I *φ*	7–9	7	10	7–8	6–7	6–8	6–8	5–6	5–6	4–5	6–7	7–8	7	7–9	8–9	6–7	7–8

Species group: *myzus* – Key characters of the group based on adult females: stigmata and tracheae present; ambulacral claws II–III present; genu I–III without setae; femur I with two setae [42].

### *Eutarsopolipus chlaenii* Katlav & Hajiqanbar n. sp. (Figs. 10–11)

urn:lsid:zoobank.org:act:25276820-D40C-4F2F-AAA2-E68575A38719

**Figure 10:**
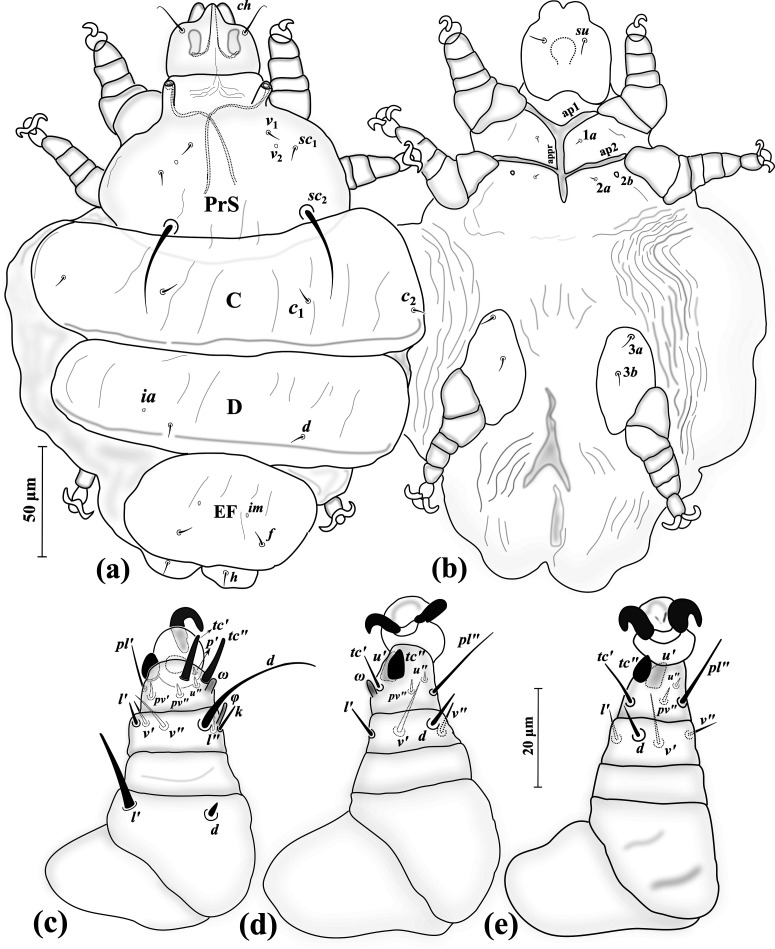
*Eutarsopolipus chlaenii* n. sp. (adult female). (a) Body dorsum; (b) body venter; (c) right leg I; (d) ventral view of tarsus I; (e) right leg II; (f) right leg III. All legs in dorsal view.

**Figure 11:**
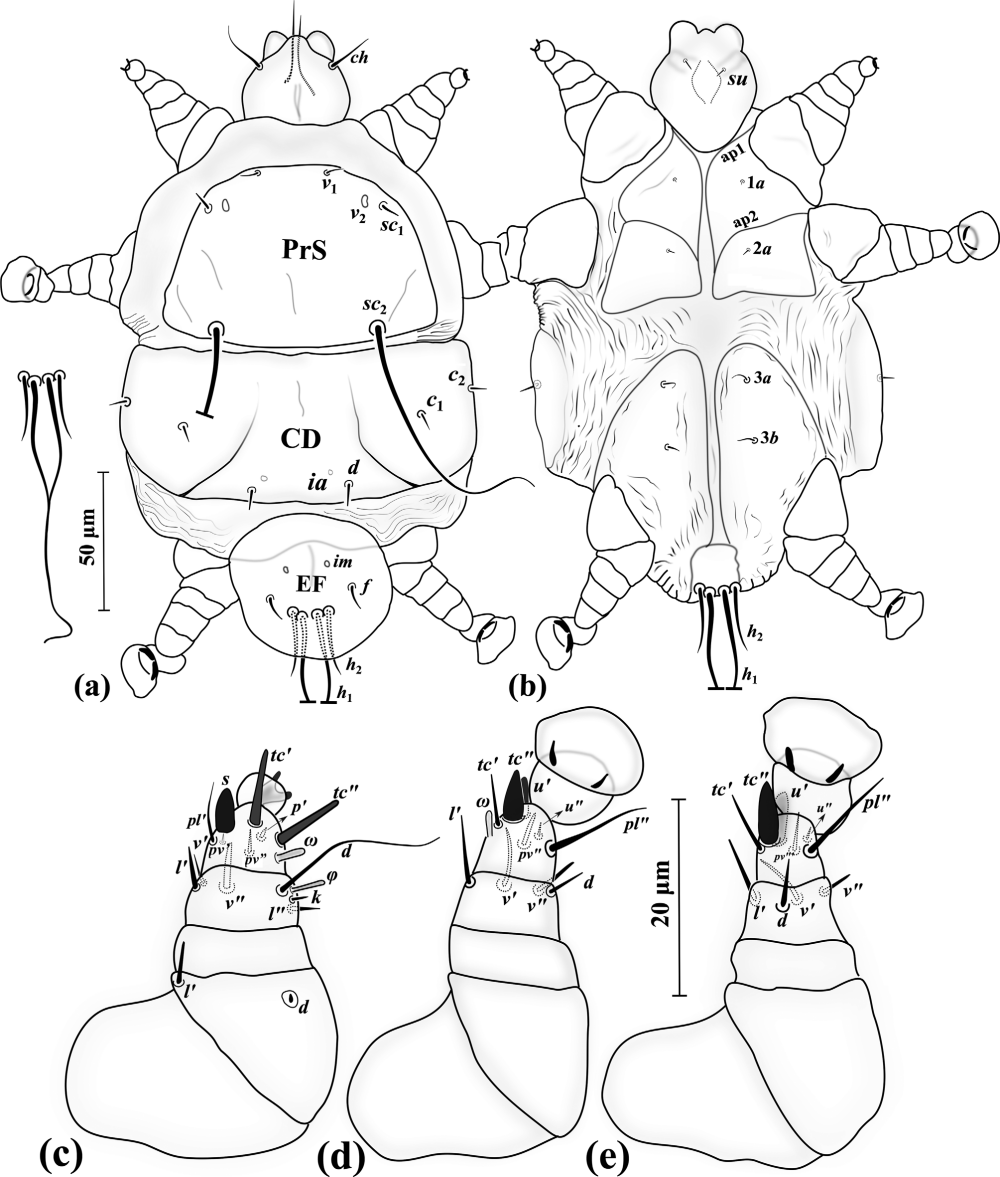
*Eutarsopolipus chlaenii* n. sp. (larval female). (a) Body dorsum; (b) body venter; (c) right leg I; (d) right leg II; (e) right leg III. All legs in dorsal view.

**Figure 12:**
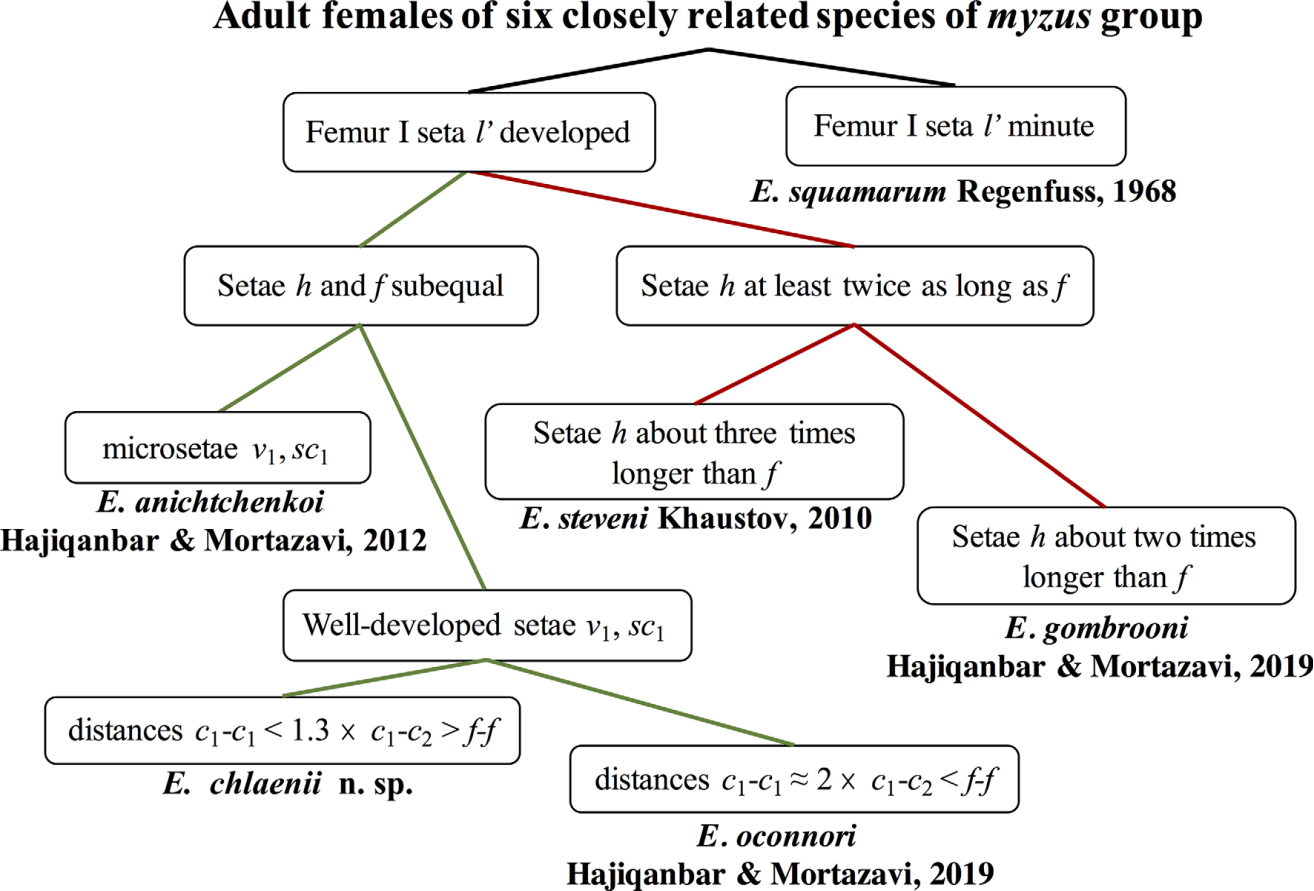
Key to closely related species of *myzus* group (based on adult females) possessing similar characters including short cheliceral stylets (<35 μm long).

**Figure 13:**
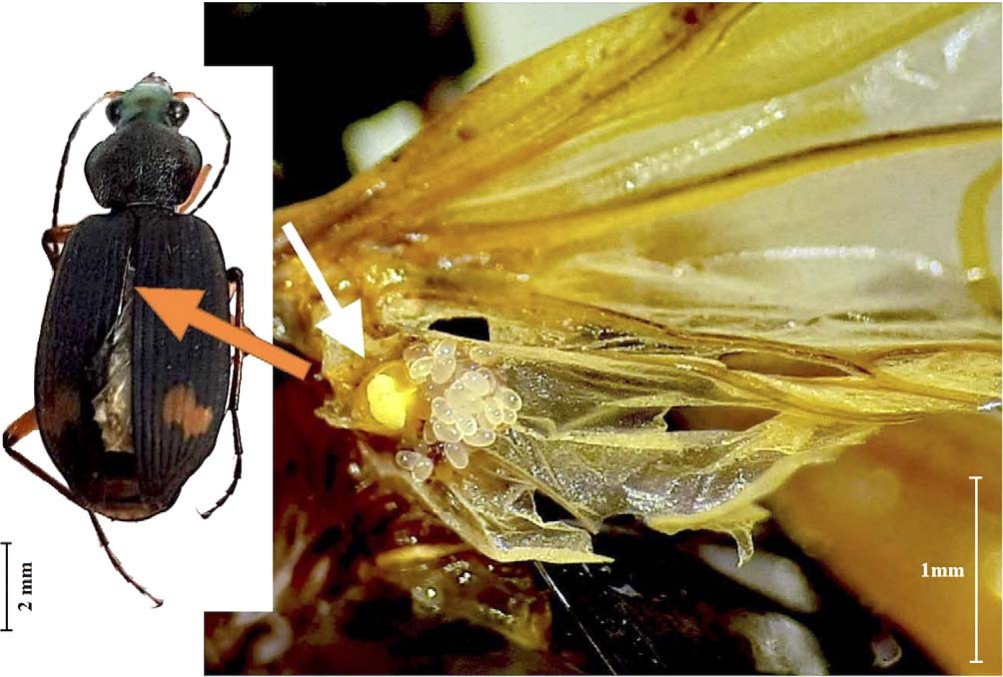
*Eutarsopolipus chlaenii* n. sp. under the elytra of the host beetle *Chlaenius flaviguttatus* Macleay, 1825, localized on the proximal portion of the host’s hindwing, with view of the mite’s engorged female (yellow colour) producing eggs (milky colour).

*Type material: Total material recovered*: ♀ (*n* = 4), larval ♀ (*n* = 16), ex. under elytra, on the base of membranous hind wing of specimens of *Chlaenius flaviguttatus* Macleay, 1825 (Coleoptera: Carabidae: Harpalinae: Chlaeniini) (Fig. 13). Three out of four collected host specimens found parasitised. Beetles specimens were collected at three independent events on 24 Feb 2020, 26 Feb 2020, and 28 Feb 2020. *Holotype*: adult female (ANIC 52-003965), ex. under elytra, on the base of membranous hind wing of *C. flaviguttatus*; Coll. Shams Paryav; 24 Feb 2020. *Paratypes*: adult female (*n* = 3), larval female (*n* = 5), same data as holotype (24 Feb 2020, 26 Feb 2020, and 28 Feb 2020).

*Type locality*: Loc. Vines Drive, Hawkesbury Campus, Western Sydney University, Richmond, NSW, 33°36′45.6″ S 150°44′40.2″ E.

*Deposition of material*: The holotype, one adult female and 2 larval female paratypes are deposited at ANIC (ANIC 52-003965-68). 1 adult female and 2 larval female paratypes are deposited at QM (QMS 117009-10, 117042). The remaining paratypes (TMU SP-20200224, 1–3), 11 non-type larval females and the host beetle specimen are deposited at AC-DE-TMU.

*Etymology*: The species epithet “*chlaenii*” refers to the generic name of the carabid host beetle *Chlaenius flaviguttatus.*

*Authorship*: Note that the authors of the new taxon are different from the authors of this paper; Article 50.1 and Recommendation 50A of International Code of Zoological Nomenclature [24].

#### Adult female (Fig. 10) (*n* = 4)

*Gnathosoma* (Figs. 10a–10b). Length 45 (43–45), width 42 (40–42); cheliceral stylets length 28 (29–30); pharynx length 12 (12–13), pharynx width 12 (12–13); *ch* 15 (15–16), pointed; *su* 6 (5–6), needle-like; distance between setae *ch–ch* 27 (27–29), *su–su* 17 (16–17).

*Idiosoma* (Figs. 10a–10b). Length 230 (225–240), width 185 (165–185).

*Idiosomal dorsum* (Fig. 10a). Respiratory system (stigmata and tracheae) present, stigmata stalked; all dorsal setae pointed; prodorsal plate (PrS) with setae *v*_1_ 6 (5–6), setae *v*_2_ vestigial, setae *sc*_1_ 5 (5–6), *sc*_2_ 42 (38–41). Plate C setae *c*_1_ 7 (6–7), *c*_2_ 5 (6–6). Plate D setae *d* 5 (5–6); cupuli *ia* evident, anterolaterad setae *d*. Plate EF setae *f* 7 (6–7); cupuli *im* evident, anterolaterad setae *f*. Plate H not evident, setae *h* 7 (7–8). Distances between setae: *v*_1_–*v*_1_ 35 (34–37), *v*_2_–*v*_2_ 42 (41–44), *v*_1_–*v*_2_ 13 (13–14), *sc*_1_–*sc*_1_ 61 (57–60), *v*_1_–*sc*_1_ 18 (18–19), *sc*_2_–*sc*_2_ 62 (58–61), *sc*_1_–*sc*_2_ 39 (38–40), *c*_1_–*c*_1_ 61 (57–64), *c*_1_–*c*_2_ 48 (42–46), *d–d* 59 (57–58), *f–f* 37 (33–36).

*Idiosomal venter* (Fig. 10b). All coxal plates smooth; all coxal setae pointed; ap1–2 and appr well developed, ap2 reaching to appr; apsej absent; coxisternal field I with setae 1*a* 3 (2–2); alveoli of vestigial setae 1*b* not evident; coxisternal field II with 2*a* 2 (2–2); alveoli of vestigial setae 2*b* evident; coxisternal field III with subequal setae 3*a* 7 (7–8) and 3*b* 7 (8–8). Distances between setae: 1*a*–1*a* 19 (20–22), 2*a*–2*a* 27 (25–26), 3*a*–3*b* 19 (24–26).

*Legs* (Figs. 10c–10e). Setal formula for legs I–III (femur-tarsus): 2-0-6(+*φ*)-8(+*ω*), 0-0-4-6(+*ω*), 0-0-4-6. Ambulacrum I with a well-developed sickle-shaped claw, ambulacrum II–III each with a pair of well-developed claws. *Leg I* (Fig. 10c): femur, *d* 3 (2–2), slightly thickened, seta *l*′ 16 (15–16) thick and blunt-ended; tibia with *φ* 5 (5–5) baculiform, *d* 28 (29–31), *l*′ 4 (4–5), *l*″ 3 (3–3), *v*′ 5 (5–6) stiff, *v*″ 9 (8–9), seta *k* 5 (5–6); tarsus I, *ω* 3 (3–4) digitiform, eupathidial setae *tc*′ 9 (8–9) and *tc*″ 10 (9–10) distinctly blunt-ended, *pl*′ 9 (8–9), setae *u*″ 2 (2–3), *pv*′ 3 (2–2) and *pv*″ 2 (2–2) subequal, seta *s* 5 (5–5) spine-like, with a blunt tip, *p*′ 1 (1–2). *Leg II.* (Fig. 10d): tibia, *d* 8 (7–8), *l*′ 7 (6–7), *v*′ 11 (10–11), *v*″ 6 (6–6); tarsus, *ω* 4 (3–4) digitiform, *tc*′ 7 (6–7), setae *u*′ 6 (6–6) and *tc*″ 5 (5–6) spine-like, *pl*″ 17 (17–18), *pv*″ 3 (3–3), *u*″ 2 (2–2). *Leg III* (Fig. 10e): tibia, setae *d* 7 (7–8), *l*′ 6 (6–7), *v*′ 11 (10–12), *v*″ 6 (5–6); tarsus, *tc*′ 10 (10–10), setae *u*′ 6 (6–7) and *tc*″ 6 (5–6) spine-like, *pl*″ 16 (15–16), *pv*″ 3 (3–3), *u*″ 2 (2–2).

#### Male (Unknown)

#### Larval female (Fig. 11) (*n* = 5)

*Gnathosoma* (Figures 11a–11b). Length 39–46, width 38–40; cheliceral stylets length 29–32; pharynx length 13–15, pharynx width 10–11; *ch* 17–19 pointed; *su* 3–4 needle-like; distance between setae *ch–ch* 22–26, *su–su* 12–14.

*Idiosoma* (Figs. 11a–11b). Length 165–195, width 125–145.

*Idiosomal dorsum* (Fig. 11a). All dorsal setae needle-like except *sc*_2_ attenuate; PrS with setae *v*_1_ 6–7, *v*_2_ vestigial, *sc*_1_ 6–7, *sc*_2_ 95–98. Plate C setae *c*_1_ 5–6, *c*_2_ 5–6. Plate D setae *d* 6–7; cupuli *ia* anterolaterad setae *d*. Plate EF setae *f* 7–8; cupuli *im* anterolaterad setae *f*. Plate H situated ventrally with setae *h*_1_ 97–101 and *h*_2_ 22–24. Distances between setae: *v*_1_–*v*_1_ 24–26, *v*_2_–*v*_2_ 44–47, *v*_1_–*v*_2_ 12–14, *sc*_1_–*sc*_1_ 59–62, *v*_1_–*sc*_1_ 20–22, *sc*_2_–*sc*_2_ 57–58, *sc*_1_–*sc*_2_ 40–42, *c*_1_–*c*_1_ 83–86, *c*_1_–*c*_2_ 21–23, *d–d* 34–36, *f–f* 28–29.

*Idiosomal venter* (Fig. 11b). All coxal plates smooth; all coxal setae tiny and pointed; ap1–2 and apsej evident; coxisternal fields I–II each divided from its pair, with setae 1*a* 1–1; alveoli of setae 1*b* not evident; coxisternal field II with 2*a* 2–3; alveoli of setae 2*b* not evident; coxisternal field III widened, with setae 3*a* 7–9 and 3*b* 7–8 subequal. Distances between setae: 1*a*–1*a* 22–34, 2*a*–2*a* 28–30, 3*a*–3*b* 21–22.

*Legs* (Figs. 11c–11e). Setal formula for legs I–III (femur-tarsus): 2-0-6(+*φ*)-7(+*ω*), 0-0-4-6(+*ω*), 0-0-4-6. Ambulacrum I with a small bifid claw, ambulacrum II–III each with a pair of tiny claws. *Leg I* (Fig. 11c): femur, *d* microseta, seta *l*′ 4–5 stiff; tibia, *φ* 3–4 baculiform, *d* 17–21, seta *l*′ 4–5 slightly thickened, *l*″ 2–3, *v*′ 3–4, seta *v*″ 4–5 slightly thickened and blunt-ended, seta *k* 2–3; tarsus, *ω* 3–4 digitiform, eupathidial setae *tc*′ 7–8 and *tc*″ 7–8 subequal, distinctly blunt-ended, *pl*′ 6–6, setae *pv*′ 1–2, *pv*″ 2–3, seta *s* 4–5 blunt spur-like, *p*′ 1–1; *u*″ not visible. *Leg II.* (Fig. 11d): tibia, *d* 4–5, *l*′ 6–7, *v*′ 6–7, *v*″ 4–6; tarsus, *ω* 2–3 digitiform, *tc*′ 5–7, setae *u*′ 4–5 and *tc*″ 5–6 blunt spur-like, *pl*″ 10–12, *pv*″ 2–2, *u*″ 2–2. *Leg III* (Fig. 11e): tibia, *d* 5–6, *l*′ 6–7, *v*′ 6–8, *v*″ 3–5; tarsus, *tc*′ 6–7, setae *u*′ 5–6 and *tc*″ 6–7 blunt spur-like, *pl*″ 10–13, *pv*″ 3–4, *u*″ 1–1.

#### Differential diagnosis

The new species belongs to a subgroup of the *myzus* species group that shares a combination of the following characters in adult females: ambulacrum I claw well-developed, idiosoma without lateral bulges or posteriorly without wrinkled lobes, shield C not divided, femur I seta *l*′ developed (not microseta), and cheliceral stylets less than 35 μm long [13]. This assemblage includes *E. chlaenii* n. sp. and four other species: *E*. *steveni* Khaustov, 2010, *E. anichtchenkoi*, Hajiqanbar & Mortazavi, 2012, *E*. *gombrooni* Hajiqanbar & Mortazavi, 2019, and *E*. *oconnori* Hajiqanbar & Mortazavi, 2019. Among these species, *E. chlaenii* n. sp. is more similar to *E. anichtchenkoi* and *E*. *oconnori* by having setae *h* and *f* subequal. However, it is readily distinguishable from *E. anichtchenkoi* by having developed setae *v*_1_, *sc*_1_, 1*a* and 2*a* (adult female with microsetae *v*_1_, *sc*_1_, 1*a* and 2*a* in *E. anichtchenkoi*), *sc*_2_ almost five times longer than *h*_1_ (adult female with *sc*_2_ at least nine times longer than *h*_1_ in *E. anichtchenkoi*) and tarsus III with six setae (tarsus III with seven setae in *E. anichtchenkoi*). *Eutarsopolipus chlaenii* n. sp. also differs from *E*. *oconnori* by having shorter distances *c*_1_-*c*_1_, *d-d*, *f-f* in the adult female (64, 59, 37 *vs.* 101, 113, 86, respectively, in *E*. *oconnori*) and longer setae *su* and *h*_1_ and cheliceral stylets in larval females (101, 4, 32, *vs.* 61, m, 26, respectively, in *E*. *oconnori*). All the important characters among these five species of the *myzus* species group are compared for all life stages (excluding *E. chlaenii* n. sp. with unknown male) in Table 3. Among adult females of the *myzus* species group with a strong claw on ambulacrum I, lateral bulges or posterior wrinkled lobes and entire shield C, six species have short cheliceral stylets (less than 35 μm long). The key to this subgroup is presented in Figure 12.

**Table 3:** Comparison of selected characters (range of measurements if available) of five closely related species of the myzus species group in *Eutarsopolipus* (male is unknown for *E. chlaenii* n. sp.): *E. chlaenii* n. sp. (*Ec*), *E. steveni* (*Es*), *E. anichtchenkoi* (*Ea*), *E. gombrooni* (*Eg*), and *E. oconnori* (*Ea*).

Life stage	Female					Male				Larval female				
Character	*Ec*	*Es*	*Ea*	*Eg*	*Eo*	*Es*	*Ea*	*Eg*	*Eo*	*Ec*	*Es*	*Ea*	*Eg*	*Eo*
Gn. L.	43–45	31–34	47–50	43–50	50	21–22	26–29	24––26	25	39–46	22–24	30–35	23	26–36
Ch. S. L.	28–30	28–31	30–35	29–32	34	14–15	12–13	13–16	13	29–32	19–20	17–19	18	16–26
Setae *ch*	15–16	17–19	12–13	14–16	14	6–7	8–10	4	5	17–19	15–17	12–13	14	11–14
Setae *su*	5–6	6–7	4–6	5–7	5	3–4	2–3	2	m	3–4	7–8	2	7	m
Setae *v*_1_	5–6	5–6	m	6–7	8	3–4	m	3–4	m	6–7	3–4	6–7	5	5–7
Setae *sc*_1_	5–6	5–6	m	8	8	3–4	m	3–4	m	6–7	3–4	5–6	5	7–8
Setae *sc*_2_	38–42	26–28	37–45	18–21	45	35–37	39–45	26–31	17	95–98	41–47	78–79	43	85–93
Setae *c*_1_	6–7	6–8	4–5	5–9	9	4–5	m	m–3	m	5–6	4–5	7–9	5	6–7
Setae *c*_2_	5–6	6–7	4–5	7–8	9	4–5	m	4–5	m	5–6	4–5	6	4	6–7
Setae *d*	5–6	6–7	4–5	6–7	8	4–5	m	m–4	m	6–7	4–5	9	3	7
Setae *f*	6–7	7–8	4–5	7–8	8	4–4	m	m–3	m	7–8	6–7	9	6	5–8
Setae *h*_1_	7–8	24–26	3–4	13–16	9	–	–	–	–	97–101	70–75	140–172	89	57–61
Setae *h*_2_	–	–	–	–	–	–	–	–	–	22–24	24–27	20–21	20	12–20
Setae 1*a*	2–3	2–3	m	2–3	m	v	m	m	m	1	2	m	m	m
Setae 2*a*	2	3–4	m	3–4	m	2	m	1–2	m	2–3	3	m	2	m
Setae 3*a*	7–8	~8	4–5	8–9	9	3–4	m	3–4	m	7–9	10	3	10	5–9
Setae 3*b*	7–8	8–9	4–5	8	11	4	m	4–5	m	7–8	6	4	9	6–8
Gen. cap. L.	–	–	–	–	–	21–22	26–29	31–34	18	–	–	–	–	–
Gen. cap. W.	–	–	–	–	–	21–22	25–28	34–35	24	–	–	–	–	–
Sol. Ta I *ω*	3–4	3–4	2	3	3	3–4	3	3–4	6	3–4	3–4	2–3	~2	2–3
Sol. Ti I *φ*	5	4–5	5	4–5	5	4–5	4	3–5	8	3–4	4–5	4	4	4–5
Sol. Ta II *ω*	3–4	3–4	2	3	3	3–4	2	3–4	6	2–3	3–4	3	3	3–4
Fe I seta *d*	2–3	m	m	m	~1	m	m	m	m	m	m	m	m	m
Fe I seta *l’*	15–16	~14	12–13	11–13	13	m	~1	~1	4	4–5	m	~3	2	4–5
Ti I seta *d*	28–31	~20	~22	17–26	18	~17	~22	15–16	28	17–21	~18	~18	~20	~19

## Discussion

Among all *Eutarsopolipus*, *leytei* is apparently the most primitive group that represents the putative plesiomorphies of a well-developed tracheal system as well as retention of genual I–III setae (2-1-1) and all femoral I setae (3 setae). Conversely, the *pterostichi* group with a missing tracheal system and genual I–III setae (0-0-0), reduction of femoral I setation (2 setae) and sometimes reduction/absence of ambulacral claws [as in *E. echinatus*, 43] may be relatively more derivative than the other Australian *Eutarsopolipus* [31, 43]. However, the *myzus* group, possessing a combination of plesiomorphies (well-developed tracheal system and ambulacral claws) and some apomorphies [reduction of femoral I setation (2 setae) and absence of genual I–III setae (0-0-0)], may hold an intermediate position. It is surprising that in our study such considerable species diversity was detected in a single location following a minimal sampling effort preformed across fewer than three weeks. This may substantiate the previously held notion that Australia exhibits diverse *Eutarsopolipus* fauna with a wide gradient of morphological variations [42]. Despite a few sporadic studies on Australian *Eutarsopolipus*, six out of the ten known species groups that exist across the world (including *ochoai*, *megacheli* and *secundus*) have so far been recorded from Australia ([31, 42, 44], present study). However, the rich diversity of Australian carabid beetles may posit the idea that the current knowledge about their associated *Eutarsopolipus* mites is still in its infancy; therefore, more extensive faunistic studies in different regions could potentially lead to the discovery of enormous diversity in *Eutarsopolipus*.

With the description of *E. chlaenii*, this study reports the *myzus* group for the first time in Australia, thereby extending its distribution to Oceania, and beyond the previously recorded Holarctic, Afrotropical and Oriental realms [12, 22]. About half of the species of this group (13/25) are parasites of carabids of the genus *Chlaenius* Bonelli [12, 22]. Furthermore, the finding of *E. pulcher* n. sp. from *G. pulcher* is the second record of the *leytei* group from a native carabid of the genus *Gnathaphanus* Macleay, 1825 (tribe Harpalini). Recently, a study in the same location found another species, *E. orpheus* from under the elytra of *Gnathaphanus melbournensis* (Castelnau, 1867), probably suggesting more specific association of the *leytei* group with carabids of *Gnathaphanus.* This carabid genus is apparently native to the Australasian and Oriental regions and represents more than 15 species in Australia [4] with *G. pulcher* and *G. melbournensis* being highly abundant in eastern Australia [3]. It is interesting, however, that the only Palearctic representative of the *leytei* group, *E. dastychi*, was found from *Calathus* of the carabid tribe Sphodrini [20] which is phylogenetically diverged from the carabid tribe Harpalini. This kind of counterintuitive host range is even more profound among the *myzus* and *pterostichi* groups, both of which are associated with carabids of the two distantly related subfamilies, Harpalinae and Scaritinae [26, 42], suggesting that several episodes of host switching may have contributed to the evolution of their host associations.

Carabid beetles are generalist predators that feed on a variety of small invertebrates including important agricultural pests and thus serve as important biocontrol agents [34]. However, their ecological interactions are often hard to predict [9]. It is unknown how the parasitic role of *Eutarsopolipus* mites can shape the ecology and evolution of carabids, yet incorporation of such information may contribute to models predicting interaction networks of carabids for future biocontrol programs.

## Conflict of interest

The authors declare that they do not have any conflict of interest.
